# Epidemiological and demographic drivers of lung cancer mortality from 1990 to 2019: results from the global burden of disease study 2019

**DOI:** 10.3389/fpubh.2023.1054200

**Published:** 2023-05-05

**Authors:** Yaguang Fan, Yong Jiang, Lei Gong, Ying Wang, Zheng Su, Xuebing Li, Heng Wu, Hongli Pan, Jing Wang, Zhaowei Meng, Qinghua Zhou, Youlin Qiao

**Affiliations:** ^1^Tianjin Key Laboratory of Lung Cancer Metastasis and Tumor Microenvironment, Tianjin Lung Cancer Institute, Tianjin Medical University General Hospital, Tianjin, China; ^2^National Cancer Center/National Clinical Research Center for Cancer/Cancer Hospital, Chinese Academy of Medical Sciences and Peking Union Medical College, Beijing, China; ^3^Department of Esophageal Cancer, National Clinical Research Center for Cancer, Key Laboratory of Cancer Prevention and Therapy of Tianjin City, Tianjin Medical University Cancer Institute and Hospital, Tianjin, China; ^4^Department of Radiology, Tianjin Medical University General Hospital, Tianjin, China; ^5^Department of Tobacco Control and Prevention of Respiratory Disease, Center of Respiratory Medicine, China-Japan Friendship Hospital, Beijing, China; ^6^Department of Nuclear Medicine, Tianjin Medical University General Hospital, Tianjin, China; ^7^Sichuan Lung Cancer Institute, Sichuan Lung Cancer Center, West China Hospital, Sichuan University, Chengdu, China; ^8^Center of Global Health, School of Population Medicine and Public Health, Chinese Academy of Medical Sciences and Peking Union Medical College, Beijing, China

**Keywords:** lung cancer, global disease burden, death, mortality, population growth, aging

## Abstract

**Background:**

Understanding the effects of demographic drivers on lung cancer mortality trends is critical for lung cancer control. We have examined the drivers of lung cancer mortality at the global, regional, and national levels.

**Methods:**

Data on lung cancer death and mortality were extracted from the Global Burden of Disease (GBD) 2019. Estimated annual percentage change (EAPC) in the age-standardized mortality rate (ASMR) for lung cancer and all-cause mortality were calculated to measure temporal trends in lung cancer from 1990 to 2019. Decomposition analysis was used to analyze the contributions of epidemiological and demographic drivers to lung cancer mortality.

**Results:**

Despite a non-significant decrease in ASMR [EAPC = −0.31, 95% confidence interval (CI): −1.1 to 0.49], the number of deaths from lung cancer increased by 91.8% [95% uncertainty interval (UI): 74.5–109.0%] between 1990 and 2019. This increase was due to the changes in the number of deaths attributable to population aging (59.6%), population growth (56.7%), and non-GBD risks (3.49%) compared with 1990 data. Conversely, the number of lung cancer deaths due to GBD risks decreased by 19.8%, mainly due to tobacco (−12.66%), occupational risks (−3.52%), and air pollution (−3.47%). More lung cancer deaths (1.83%) were observed in most regions, which were due to high fasting plasma glucose levels. The temporal trend of lung cancer ASMR and the patterns of demographic drivers varied by region and gender. Significant associations were observed between the contributions of population growth, GBD risks and non-GBD risks (negative), population aging (positive), and ASMR in 1990, the sociodemographic index (SDI), and the human development index (HDI) in 2019.

**Conclusion:**

Population aging and population growth increased global lung cancer deaths from 1990 to 2019, despite a decrease in age-specific lung cancer death rates due to GBD risks in most regions. A tailored strategy is needed to reduce the increasing burden of lung cancer due to outpacing demographic drivers of epidemiological change globally and in most regions, taking into account region- or gender-specific risk patterns.

## Introduction

Lung cancer is the leading cause of cancer-related death worldwide, with an estimated 1.8 million deaths in 2018 (1). It is estimated that the number of lung cancer deaths worldwide will increase by 67% by 2040 (1). Consequently, reducing mortality from lung cancer, one of the main causes of non-communicable diseases, would help reduce premature mortality from non-communicable diseases by one-third by 2030, which is one of the United Nations Sustainable Development Goals (2).

Several factors are associated with an increased risk of lung cancer. Tobacco use is the leading cause of lung cancer (3). Therefore, lung cancer incidence and mortality are closely related to smoking habits. Other factors, such as air pollution, occupational exposure, and poor diet, may interact with tobacco smoking to influence the epidemiology of lung cancer (4–6). Changes in the lung cancer burden due to these risk factors have contributed the most to the decline in lung cancer ASMR from 1990 to 2019 (7). However, the absolute number of lung cancer deaths is increasing. This increase is due to the combined effects of population growth, aging, and epidemiological changes in lung cancer (8, 9). Approximately 0.38 million lung cancer deaths worldwide are attributable to population aging (8). Additionally, the trends and drivers of lung cancer mortality vary substantially by SDI (9). To plan for the future of the healthcare system, it is important to decipher the impact of population growth, aging, and age-specific lung cancer mortality due to risk factors, individually or in combination, on lung cancer mortality trends.

The GBD study provides a unique opportunity to assess long-term trends in the global, regional, and national burden of lung cancer attributable to specific risks or a combination of all risks included in the GBD (7, 10–12). However, little is known about trends in lung cancer mortality in the context of major demographic changes (13). In this study, we compared trends in all-cause lung cancer and ASMR and decomposed the contribution of demographic drivers and changes in age-specific lung cancer mortality due to risks within and outside GBD 2019. Our goal was to gain a better understanding of the role and relative magnitude of these demographic and epidemiological drivers. We also examined the relationship between changes in these drivers and ASMR in 1990 and SDI and HDI in 2019.

## Materials and methods

### Overview of GBD 2019

Age-specific, all-age, and age-standardized lung cancer mortality by various risk factors were derived from GBD 2019 (14). The GBD 2019 provides annual burden of disease estimates for 369 diseases and injuries for 87 risk factors and combinations of risk factors in 204 countries and territories from 1990 to 2019. The detailed methodology of the GBD 2019 estimation process, including the risk factor hierarchy, determination of the inclusion of risk-outcome pairs, estimation of the relative risk as a function of exposure for each risk-outcome pair, estimation of the distribution of exposure for each risk, the estimation of the population attributable fraction and burden by age-gender-location-year, and the summary exposure value, has been described in detail elsewhere (14).

### Data input

We chose “tracheal, bronchial, and lung cancer” (International Classification of Diseases-10 codes: C33–C34.9, D02.1–D02.3, D14.2–D14.3, and D38.1) as the outcome. The original data on lung cancer burden by site and year in the GBD study were obtained from the individual cancer registry system and the aggregated database of cancer registries, including the Cancer Incidence in Five Continents (CI5), the Surveillance, Epidemiology, and End Results (SEER) database, and the Nordic Cancer Registries database (NORDCAN) (10). Sources of lung cancer mortality data include vital registration, verbal autopsy, and cancer registries (15). This study complies with the Guidelines for Accurate and Transparent Health Estimates Reporting (GATHER) statement (16).

### Risk hierarchy for lung cancer

Global Burden of Disease risk factors are classified into a four-level risk hierarchy, ranging from general risk categories (behavioral, environmental, occupational, and metabolic; level 1) to the most specific factors (such as household air pollution from solid fuels; level 4). Level 2 risk factors for lung cancer, in order of attributable fraction from highest to lowest, are tobacco use, air pollution, occupational risks, high fasting plasma glucose, other environmental factors, and dietary risks (17). However, GBD risk factors did not account for all lung cancer deaths. Therefore, this study also analyzed lung cancer deaths due to non-GBD 2019 risks, which were considered risk-deleted deaths (18, 19). The risk-deleted rate is the death rate that would be observed if all risk factors included in the GBD 2019 were removed. It was calculated as the death rate multiplied by 1 minus the proportion attributable to all-risk in the population fraction at each site (17, 18). Changes in risk-deleted rates could reflect changes in risks or risk-outcome pairs that are not included in the GBD 2019, or changes in other factors such as improved treatments. Finally, we combined all GBD risks and non-GBD risks as “all-cause” lung cancer, which fully accounted for all lung cancer deaths.

### Geographical units of analysis

A total of 204 countries and territories were grouped into 21 regions. These countries and territories were also categorized into five regions in terms of SDI: low, low-middle, middle, high-middle, and high SDI regions. The SDI is a composite indicator of the level of development, originally constructed for GBD 2015 based on the total fertility rate under age 25, the average educational attainment of those aged 15 and over, and the lagged distribution of *per capita* income (20).

### Statistical analysis

Data on deaths, all ages, and age-standardized mortality from lung cancer were expressed as numbers and 95% UIs. The 95% UIs were calculated by taking 1,000 samples from the posterior distribution of each step in the modeling process and reported as 2.5- and 97.5-values for each estimate. They were obtained directly from the Global Health Data Exchange website. The estimated EAPC and its 95% CI were used to estimate trends in lung cancer mortality for all age groups and ASMR from 1990 to 2019 (12).

To analyze the drivers of lung cancer mortality, we used age- and risk-specific mortality data from the GBD 2019 to perform decomposition analyses using Das Gupta’s method (21, 22). This method develops several counterfactual scenarios and calculates the impact of each factor on changes from current levels, assuming that all other factors except the factor under consideration remain the same over the study period. In this study, two sets of decomposition analyses were performed. First, we decomposed the changes in lung cancer mortality over time into four components: (1) population growth, (2) population aging, changes in the age structures of the population, (3) all risks in the GBD 2019, and (4) non-GBD risks. The second decomposition analysis further divided the changes in all GBD risks into level 2 risk factors: tobacco, air pollution, occupational risks, high fasting plasma glucose, other environmental factors, and dietary risks. The contribution of changes in exposure to each level 2 risk was scaled to the all-risk effect (18). The relative contributions of these drivers to the change in the number of lung cancer deaths were calculated, using the number of deaths in 1990 as a reference. A positive contribution indicated an increase in lung cancer deaths. GBD risks and non-GBD risks present the epidemiological profile of GBD lung cancer risks that cannot be explained by population growth or population aging.

Based on the results of the decomposition analysis at the regional level, hierarchical cluster analysis was used to group the patterns of demographic and epidemiological drivers of lung cancer deaths from 1990 to 2019. Finally, Spearman’s correlation analysis was performed to examine the relationship between the contributions of population growth, aging, all GBD risk, individual level 2 risk, and the ASMR of lung cancer in 1990 and the SDI and HDI in 2019. The ASMR of lung cancer in 1990 reflects the disease reservoir at baseline, and the SDI/HDI in 2019 can serve as surrogates for the development status of each country. The HDI is a measure used by the United Nations and consists of three components: life expectancy, average income per person, and education level (23). The estimated global, regional, and national populations in 1990 and 2019 used for the decomposition analysis were GBD population standards (20). All statistics and visualizations were generated using R software version 4.1.3 and Stata 16.

## Results

### Global, regional, and national trends in lung cancer mortality

**Figure 1:**
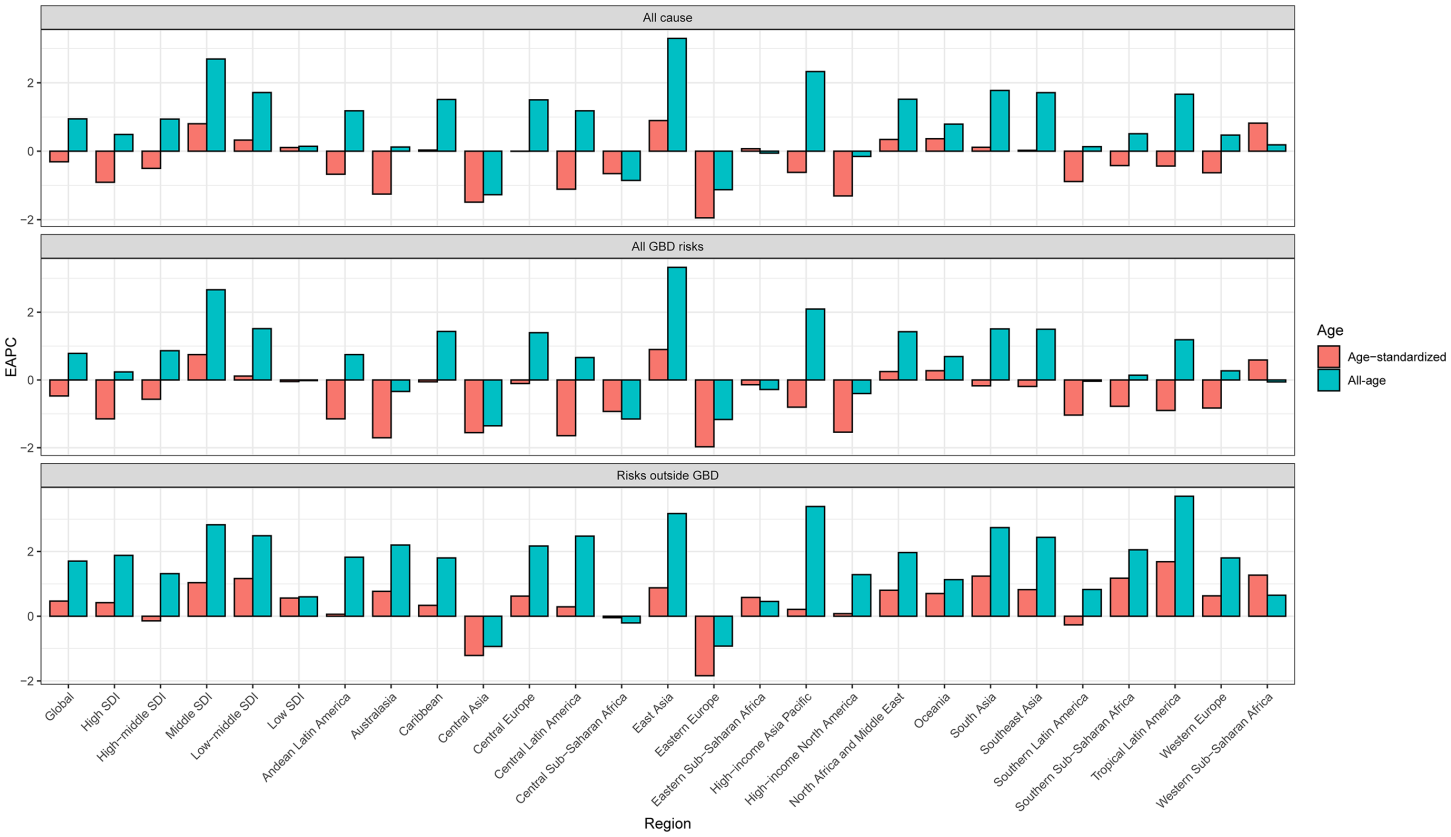
Global and regional EAPC of all-cause and age-standardized lung cancer mortality from 1990 to 2019.

Globally, the number of lung cancer deaths increased from 1.07 million (95% UI: 1.02–1.11) in 1990 to 2.04 million (95% UI: 1.88–2.19) in 2019, corresponding to an EAPC of 0.94 (95% CI: 0.07–1.83) for all-cause mortality (Table 1; Figure 1; Supplementary Table S1). In contrast, the EAPC of −0.31 (95% CI: −1.1-0.49) indicated a non-significant decreasing trend for ASMR. Similar trends were observed for lung cancer due to all GBD risks. In contrast, there was an increasing trend, although not significant, for lung cancer ASMR due to non-GBD risks (EAPC = 0.47, 95% CI: −1.44-2.42).

**Table 1:** Deaths and mortality of lung cancer in 1990 and 2019 by region and risk factor.

Characteristics	1990			2019		
	Deaths (95%UI)	All-cause mortality (95%UI)	ASMR (95%UI)	Deaths (95%UI)	All-cause mortality (95%UI)	ASMR (95%UI)
Global	1,065,139(1019217–1,117,181)	19.91(19.05–20.88)	27.3(26.03–28.59)	2,042,640(1879241–2,193,269)	26.4(24.29–28.35)	25.18(23.16–27.01)
SDI status						
High SDI	395,555(383434–402,047)	48.12(46.65–48.91)	37.76(36.62–38.36)	577,535(533701–603,009)	56.99(52.67–59.5)	29.78(27.82–30.96)
High-middle SDI	362,843(344098–382,496)	31.54(29.91–33.25)	33.57(31.83–35.39)	614,151(559106–669,923)	42.94(39.09–46.83)	29.9(27.22–32.6)
Middle SDI	217,710(199427–238,654)	12.68(11.62–13.9)	15.54(9.86–29.29)	629,956(550778–711,832)	26.29(22.98–29.7)	26.3(23.01–29.66)
Low-middle SDI	68,291(61274–77,261)	6.05(5.42–6.84)	11.88(10.59–13.44)	174,079(158066–189,936)	9.87(8.96–10.77)	13.18(12.02–14.35)
Low SDI	20,247(16445–24,835)	3.83(3.11–4.7)	9.07(7.31–11.03)	45,976(39578–53,936)	4.07(3.51–4.78)	9.41(8.14–10.91)
Region						
Andean Latin America	2,734(2382–3,089)	7.16(6.24–8.09)	13.79(12.03–15.58)	6,241(5044–7,618)	9.81(7.93–11.98)	11.39(9.22–13.89)
Australasia	7,950(7691–8,161)	39.21(37.93–40.25)	33.45(32.33–34.34)	12,036(11086–12,767)	41.41(38.14–43.93)	23.75(22.05–25.13)
Caribbean	5,791(5482–6,125)	16.42(15.54–17.37)	22.59(21.33–23.88)	11,233(9633–13,109)	23.82(20.42–27.79)	21.67(18.6–25.29)
Central Asia	14,132(13710–14,556)	20.4(19.79–21.02)	29.08(28.22–29.92)	13,990(12661–15,452)	14.96(13.54–16.52)	19.04(17.29–20.86)
Central Europe	58,091(56981–59,005)	47.24(46.34–47.98)	38.76(37.96–39.37)	81,333(71419–92,224)	71.21(62.53–80.74)	38.26(33.62–43.52)
Central Latin America	12,036(11611–12,312)	7.33(7.07–7.5)	15.12(14.48–15.51)	27,246(23277–31,853)	10.9(9.31–12.74)	11.8(10.1–13.77)
Central Sub-Saharan Africa	3,420(2042–6,636)	6.16(3.68–11.95)	21.58(19.64–23.66)	6,890(4472–11,721)	5.24(3.4–8.91)	13.47(8.9–22.27)
East Asia	264,700(229034–303,023)	21.61(18.69–24.73)	30.99(27.11–35.21)	778,387(658108–907,254)	52.87(44.7–61.63)	38.38(32.72–44.57)
Eastern Europe	98,124(95551–99,904)	43.32(42.18–44.11)	34.08(33.15–34.73)	78,716(69568–87,982)	37.49(33.13–41.9)	22.72(20.09–25.34)
Eastern Sub-Saharan Africa	5,210(4311–6,318)	2.74(2.27–3.32)	7.36(6.09–8.82)	11,445(9629–13,857)	2.78(2.34–3.37)	7.62(6.45–9.15)
High-income Asia Pacific	50,372(48202–51,474)	29.03(27.78–29.67)	25.58(24.33–26.19)	110,970(96055–119,283)	59.25(51.29–63.69)	22.17(19.73–23.56)
High-income North America	171,315(165516–174,649)	60.98(58.92–62.17)	49.05(47.5–49.95)	230,304(216011–239,359)	63.17(59.25–65.66)	35.9(33.82–37.29)
North Africa and Middle East	29,545(24837–35,347)	8.56(7.2–10.24)	17.54(14.85–20.95)	72,510(64112–81,925)	11.91(10.53–13.46)	17.55(15.56–19.82)
Oceania	572(442–818)	8.85(6.83–12.64)	20.55(15.99–29.1)	1,489(1126–2,114)	11.22(8.48–15.92)	22.9(17.76–31.99)
South Asia	42,859(36582–49,564)	3.9(3.33–4.52)	8.11(6.85–9.34)	119,644(101195–137,368)	6.63(5.61–7.61)	8.75(7.44–10.05)
Southeast Asia	53,856(48786–59,204)	11.54(10.45–12.68)	21.69(19.86–23.71)	134,566(112781–155,885)	19.97(16.74–23.14)	23(19.25–26.63)
Southern Latin America	13,751(13357–14,111)	27.76(26.96–28.48)	29.7(28.82–30.47)	19,638(18456–20,637)	29.42(27.65–30.91)	23.46(22.04–24.64)
Southern Sub-Saharan Africa	5,595(4739–7,137)	10.66(9.03–13.6)	20.59(17.39–26.41)	10,473(9520–11,630)	13.33(12.12–14.8)	19.22(17.53–21.2)
Tropical Latin America	15,902(15393–16,322)	10.4(10.07–10.68)	17.86(17.19–18.39)	37,868(35433–39,823)	16.94(15.85–17.81)	15.8(14.74–16.63)
Western Europe	202,255(196989–205,380)	52.59(51.22–53.4)	35.18(34.31–35.72)	260,784(243990–270,442)	59.77(55.92–61.98)	28.99(27.43–29.97)
Western Sub-Saharan Africa	6,927(5767–8,293)	3.6(2.99–4.31)	8.35(7.04–9.95)	16,873(14180–19,858)	3.7(3.11–4.35)	9.96(8.48–11.62)
Risk factors						
All GBD risks	896,579(853078–948,031)	16.76(15.95–17.72)	20.2(18.41–21.95)	1,640,137(1495960–1,782,594)	21.2(19.33–23.04)	20.2(18.41–21.95)
Tobacco	770,252(734843–812,630)	14.4(13.74–15.19)	16.71(15.27–18.13)	1,358,820(1242586–1,472,977)	17.56(16.06–19.04)	16.71(15.27–18.13)
Air pollution	219,952(164775–278,637)	4.11(3.08–5.21)	4.75(3.53–6)	387,445(288043–490,056)	5.01(3.72–6.33)	4.75(3.53–6)
Occupational risks	169,840(129542–209,903)	3.17(2.42–3.92)	3.62(2.76–4.48)	289,793(221524–359,238)	3.75(2.86–4.64)	3.62(2.76–4.48)
Other environmental risks	47,566(9344–91,558)	0.89(0.17–1.71)	1.03(0.2–2)	83,703(16498–162,454)	1.08(0.21–2.1)	1.03(0.2–2)
Dietary risks	48,387(15858–71,838)	0.9(0.3–1.34)	0.95(0.28–1.42)	77,192(22552–115,145)	1(0.29–1.49)	0.95(0.28–1.42)
High fast plasma glucose	67,264(14645–151,218)	1.26(0.27–2.83)	2.22(0.53–4.83)	179,049(42685–389,384)	2.31(0.55–5.03)	2.22(0.53–4.83)

All five SDI regions showed an upward trend in lung cancer mortality for all ages. However, declining trends in ASMR were observed in the high and high-middle SDI regions, with EAPCs of −0.91 (95% CI: −1.61 to −0.2) and − 0.50 (95% CI:-1.22–0.23), respectively. Similar trends were observed for lung cancer mortality due to all GBD risks (Figure 1; Supplementary Table S1). With the exception of the high-middle SDI region, the other four SDI regions showed an increasing trend in the ASMR for lung cancer due to non-GBD risks.

For all-cause lung cancer or lung cancer due to GBD risks, the significantly increasing trends in lung cancer mortality for all ages and ASMR were found only in East Asia, with the corresponding EAPCs of 3.30 (95% CI: 2.56–4.04) and 0.90 (95% CI: 0.20–1.59), respectively. Conversely, significant decreasing trends for both all-cause lung cancer mortality and ASMR were observed in Central Asia and Eastern Europe. In high-income North America, southern Latin America, and Australasia, there was a significant downward trend in the EAPC of ASMR for both all-cause lung cancer and lung cancer due to GBD risks, whereas in Western Europe there was a significant decrease in ASMR only for lung cancer due to all GBD risks. All-cause lung cancer mortality increased significantly between 1990 to 2019 in high-income Asia and the Pacific, South Asia, tropical Latin America, North Africa and the Middle East, the Caribbean, and central Europe, but no significant increase in ASMR was observed in these regions. With the exception of Central Asia, Eastern Europe, and southern Latin America, the other regions showed increasing trends in both all-cause mortality and ASMR for lung cancer due to non-GBD risks (Figure 1; Supplementary Table S1).

### Drivers of trends in lung cancer deaths from 1990 to 2019

**Figure 2:**
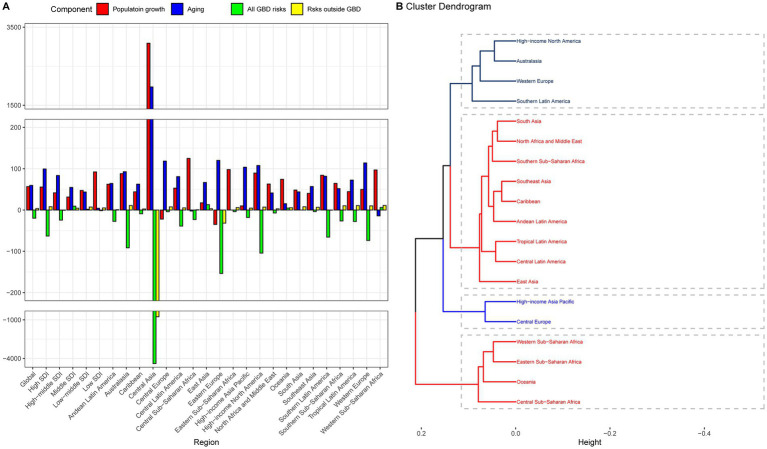
Contribution of changes in population growth, population aging, and age-specific lung cancer mortality rates from GBD risks and non-GBD risks to changes in lung cancer mortality, 1990–2019, with 1990 as the reference year. **(A)** Decomposition analysis at the regional level, **(B)** Hierarchy cluster analysis to group the patterns of demographic and epidemiologic drivers of lung cancer mortality. The regions in the five dotted boxes represent the five groups in Table 2 that had an increase in lung cancer deaths from 1990 to 2019 but had different patterns of demographic and epidemiologic changes in lung cancer mortality.

From 1990 to 2019, the excess number of lung cancer deaths was nearly 0.98 million, an increase of 91.8% (95% UI: 74.5–109.0%), although the number of lung cancer deaths attributable to GBD risks decreased by −19.8% compared with that in 2019 (Table 2; Figure 2A). This increase was driven by the changes in the number of deaths attributable to population aging (59.6% increase compared with 1990), population growth (56.7% increase compared with 1990), and non-GBD risks (3.49% increase compared with 1990). For the SDI region, the middle SDI region had the largest increase in both the number of lung cancer deaths and the percentage change in lung cancer mortality due to changes in age-specific lung cancer mortality from all GBD risks.

**Table 2:** Patterns of demographic and epidemiologic change in lung cancer mortality (1990–2019).

Location	Absolute change	Percent change (%)	Drivers of percent change (%)				Group	Group characteristics
			Population growth	Aging	All GBD risks	Non-GBD risks		
Global	977,500	91.77	56.73	59.58	−19.8	3.49	-	
High SDI	181,980	46.01	55.64	99.32	−62.94	7.98	-	
High-middle SDI	251,308	69.26	41.68	83.6	−24.53	−0.75	-	
Middle SDI	412,246	189.36	31.56	54.63	9.36	4.45	-	
Low-middle SDI	105,787	154.91	47.36	43.51	1.9	7.23	-	
Low SDI	25,728	127.07	92.2	4.25	−1.53	5.09	-	
Central Asia	−143	−1.01	3089.18	1971.22	−4397.11	−763.29	1	Lung cancer deaths decreased
Eastern Europe	−19,407	−19.78	−34.9	120.21	−153.61	−31.69		
Western Europe	58,529	28.94	49.79	113.89	−73.72	10.04	2	Lung cancer deaths increased; Population growth: Significantly increased Aging: Significantly increased Age-specific lung cancer mortality: Significantly decreased
Southern Latin America	5,888	42.81	84.23	81.82	−65.63	−0.41		
High-income North America	58,988	34.43	89.35	107.86	−104.31	7.11		
Australasia	4,086	51.4	87.94	92.67	−91.58	10.97		
South Asia	76,786	179.16	48.28	43.87	−0.03	7.88	3	Lung cancer deaths increased; Population growth: Increased Aging: Increased Age-specific lung cancer mortality: Stable or decreased
Southern Sub-Saharan Africa	4,877	87.17	64.5	51.81	−26.55	10.24		
North Africa and the Middle East	42,965	145.42	62.96	41.42	−7.18	2.81		
Caribbean	5,442	93.98	44.03	62.62	−9.15	2.5		
Southeast Asia	80,711	149.86	40.28	56.86	−3.82	6.68		
Andean Latin America	3,507	128.29	62.44	64.28	−27.42	0.7		
Central Latin America	15,210	126.37	52.86	80.8	−38.9	5.24		
Tropical Latin America	21,967	138.14	44.51	72.4	−27.71	10.8		
Central Europe	23,242	40.01	−22.13	118.47	−3.97	7.62	5	Lung cancer deaths increased; Mostly due to aging
High-income Asia Pacific	60,598	120.3	10.03	103.6	−18.15	4.51		
Eastern Sub-Saharan Africa	6,235	119.69	98.02	0.02	−3.81	5.78	6	Lung cancer deaths increased; Mostly due to population growth
Central Sub-Saharan Africa	3,469	101.44	124.83	−2.41	−23	0.58		
Western Sub-Saharan Africa	9,947	143.59	97.05	−14.19	6.12	11.02		
Oceania	916	160.13	74.33	15.32	4.8	5.55		
East Asia	513,687	194.06	17.46	66.66	12.94	2.94	4	Lung cancer deaths increased; All drivers increased

The combined effects of population growth, population aging, and age-specific mortality from GBD risks and non-GBD risks underlie the divergent trends in total lung cancer deaths observed worldwide. Only two regions, East Asia and Eastern Europe, experienced decreases in lung cancer deaths of 10 and 20%, respectively (Table 2). To better understand these complex interactions, we classified the remaining regions into five general demographic and epidemiological patterns based on a hierarchical cluster analysis in regions with increased lung cancer deaths (Figure 2B; Table 2). Then, all 21 regions were classified into six groups. Group 1 represents regions where GBD risks contributed to a relative decrease in lung cancer deaths. Group 2 represents regions where an increase in lung cancer deaths was due to a significant increase in population growth, aging, and a significant decrease in age-specific lung cancer mortality due to GBD risks. Groups 3–5 represent regions where the increase in lung cancer deaths was mainly due to population growth and/or aging. East Asia was the only group where the number of lung cancer deaths increased due to all four drivers.

**Figure 3:**
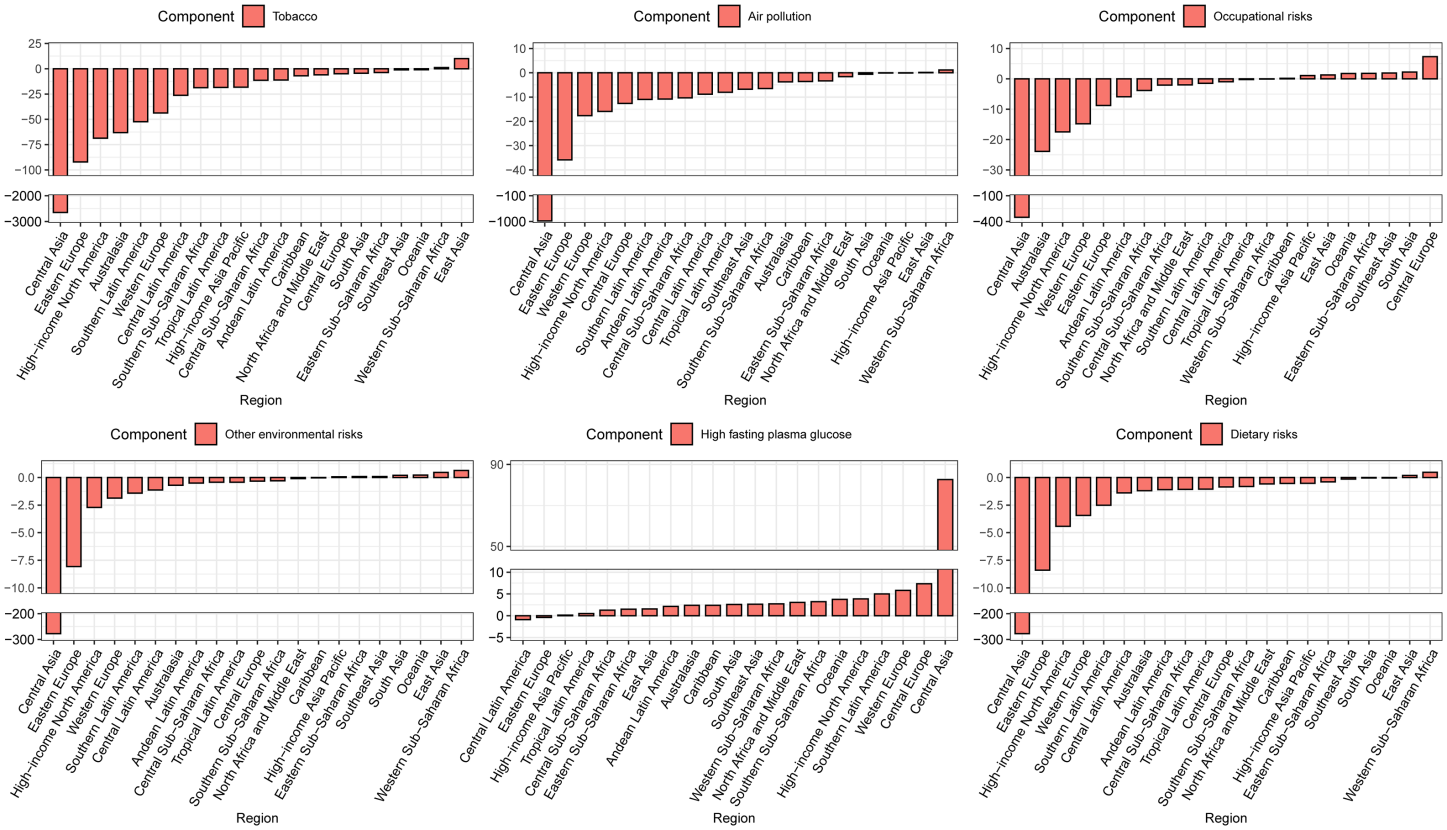
Contribution of changes in population growth, population aging, and age-specific lung cancer death rates from specific level 2 risk factors to changes in lung cancer mortality, 1990–2019 at the regional level, using 1990 as the reference year.

As shown in Supplementary Figure S1, 84 and 80% of global lung cancer deaths were attributable to GBD risks (tobacco, air pollution, occupational risks, high fasting plasma glucose, other environmental risks, and dietary risks) in 1990 and 2019, respectively. We further performed the decomposition analysis at the regional level based on six specific level 2 risk factors rather than the overall GBD risks (Figure 3; Supplementary Table S2). The decrease in lung cancer deaths was mainly due to tobacco (−12.66%), occupational risks (−3.52%), and air pollution (−3.47%). Similar to the result for all GBD risks, the regions with the largest decreases in lung cancer deaths from all GBD risks, including Central Asia, Eastern Europe, high-income North America, Australasia, Western Europe, and Southern Latin America, also had the largest decreases in lung cancer deaths from tobacco, air pollution, occupational risks, other environmental risks, and dietary risks. Conversely, these regions experienced the largest increase in lung cancer deaths due to high fasting plasma glucose.

**Figure 4:**
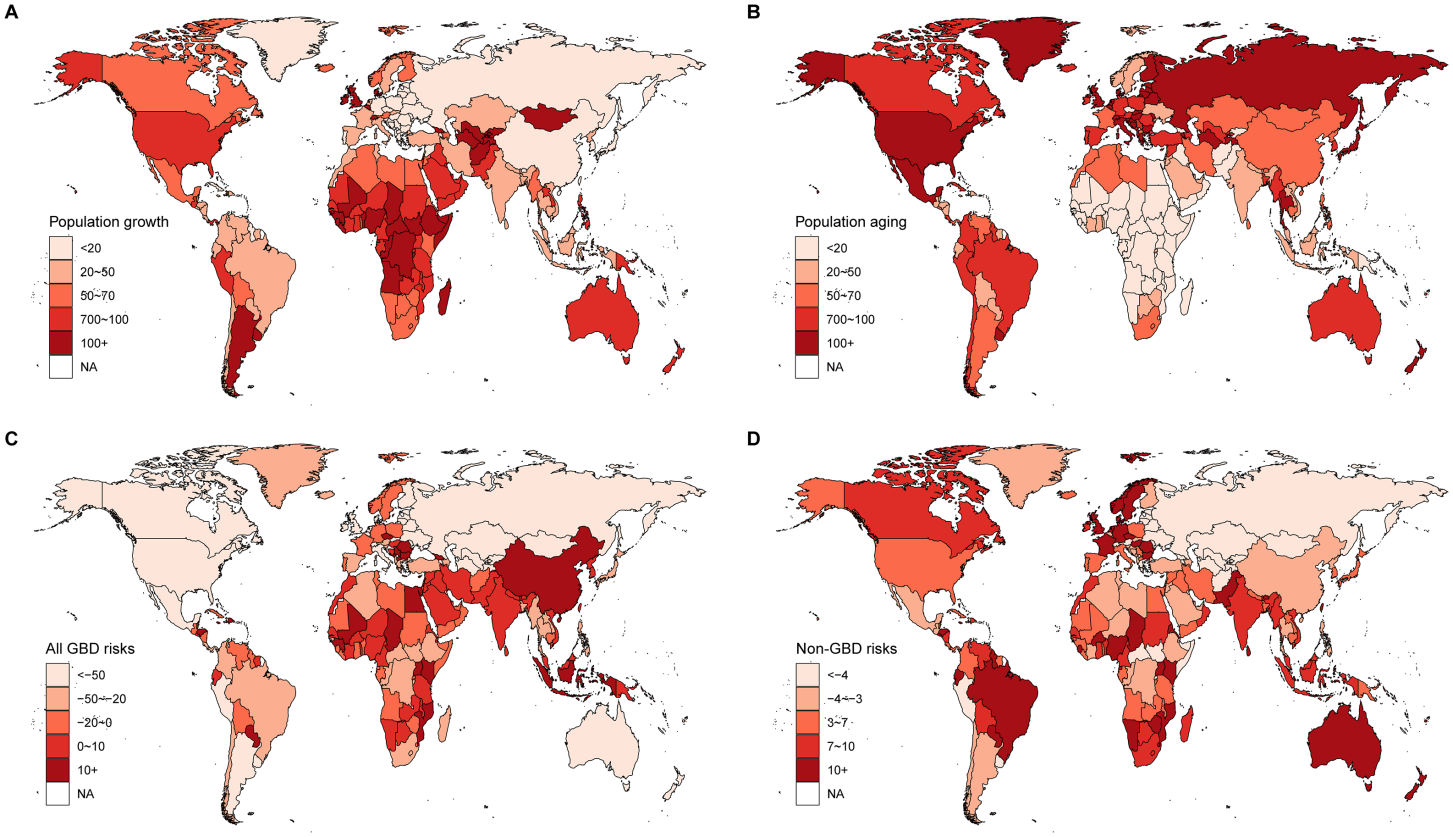
Contribution of changes in population growth, population aging, and age-specific lung cancer death rates from GBD risks and non-GBD risks to changes in lung cancer mortality, 1990–2019 at the national level, with 1990 as the reference year. **(A)** Population growth, **(B)** Population aging, **(C)** Risks in GBD 2019, **(D)** Non-GBD risks for the year 2019.

The contributions of population growth, aging, and age-specific lung cancer mortality due to GBD risks and non-GBD risks at the national level are shown in Figure 4 and Supplementary Table S3. A total of 131 countries/territories showed a decrease in lung cancer deaths due to age-specific lung cancer mortality from GBD risks, including the largest decreases in the United Kingdom (−11026.7%), Switzerland (−3221.7%), Belgium (−1466.3%), the Czech Republic (−1417.6%), and Tajikistan (−1305.4%). These countries also had the highest percentage changes in lung cancer deaths due to population growth and aging. The contributions of tobacco, air pollution, occupational risks, high fasting plasma glucose, other environmental risks, and dietary risks to the changes in lung cancer deaths are shown in Supplementary Figure S2 and Supplementary Table S4.

### Gender differences in the drivers of lung cancer deaths from 1990 to 2019

**Figure 5:**
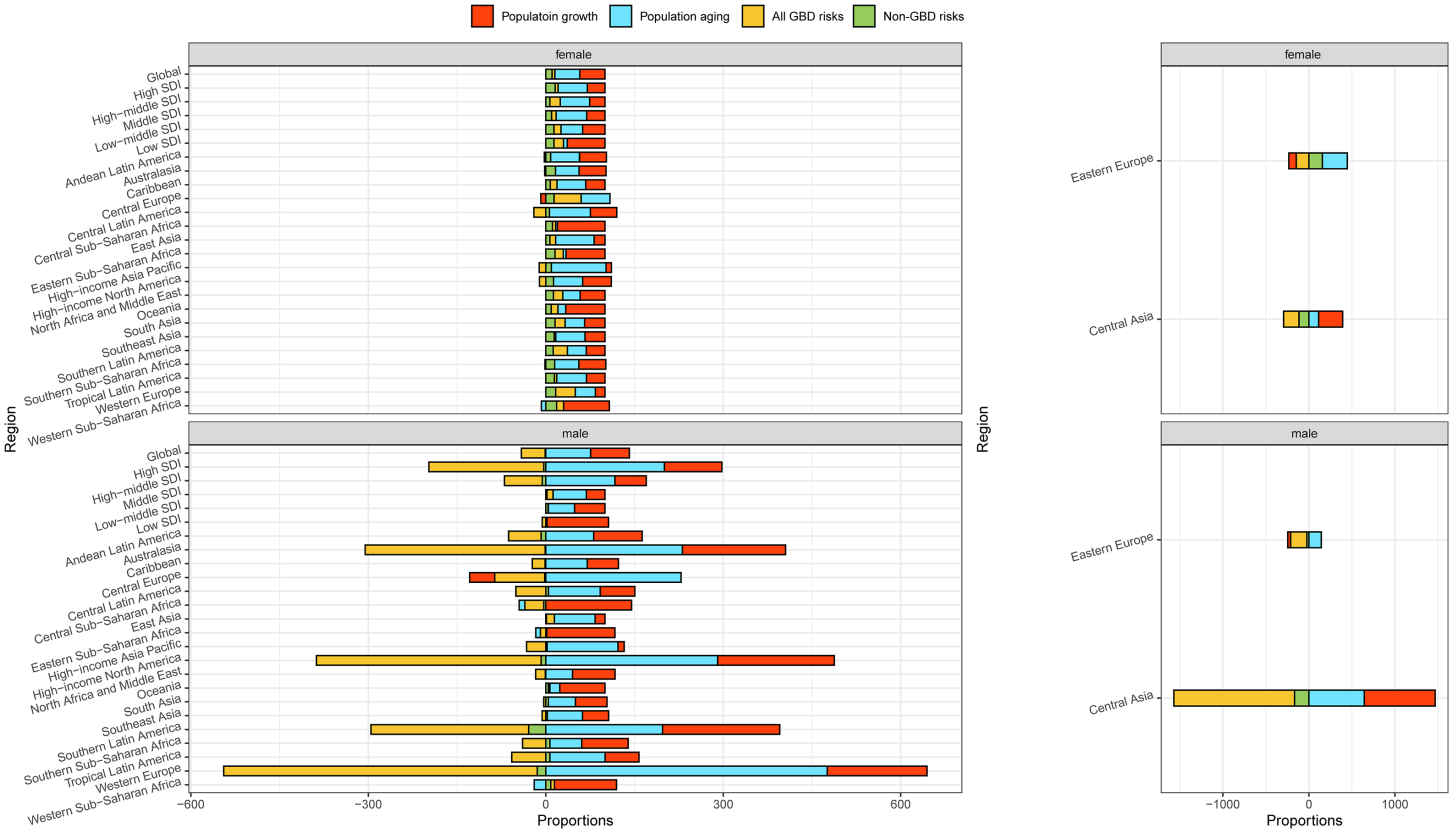
Contribution of changes in population growth, population aging, and age-specific lung cancer death rates from specific level 2 risk factors to changes in lung cancer mortality, 1990–2019 at the regional level by gender, using 1990 as the reference year.

We repeated the decomposition analysis by gender at the global and regional levels (Figure 5; Supplementary Table S5). The magnitude of the increase in lung cancer deaths from 1990 to 2019 was higher in women than in men, globally and in all regions. Compared with the decrease in the number of lung cancer deaths due to all GBD risks and non-GBD risks in men (−40.39% and − 1.03%, respectively), the number of lung cancer deaths due to these two drivers is still increasing in women worldwide (4.79 and 10.63%, respectively). The increase in the number of lung cancer deaths due to population growth and aging was remarkably lower in women (42.49 and 42.09%, respectively) than in men (65.65 and 75.78%, respectively). These patterns were more evident in the high and high-middle SDI regions. Population growth was the main driver of the increase in lung cancer deaths in the low SDI region for both men and women, and this proportion was also higher for men (103.92%) than for women (63.69%). Furthermore, compared with the decrease in lung cancer deaths from 1990 to 2019 in men, 10.63% of the excess lung cancer deaths in 2019 in women worldwide were attributed to non-GBD risks. A similar pattern was observed in most regions, except East Asia and the middle SDI region. Geographical differences in epidemiological and demographic drivers were observed for both men and women.

As shown in Supplementary Figure S3, when the all GBD risks were divided into six level 2 risks, the contributions of specific level 2 risk factors to lung cancer deaths are higher in women than men worldwide mostly. In general, the contributions of specific level 2 risk factors to lung cancer deaths are higher for women than for men worldwide. This is particularly true for tobacco. Tobacco-related lung cancer deaths decreased by −40.39% in men but increased by 4.79% in women between 1990 and 2019.

### Influential factors for different drivers of lung cancer deaths

**Figure 6:**
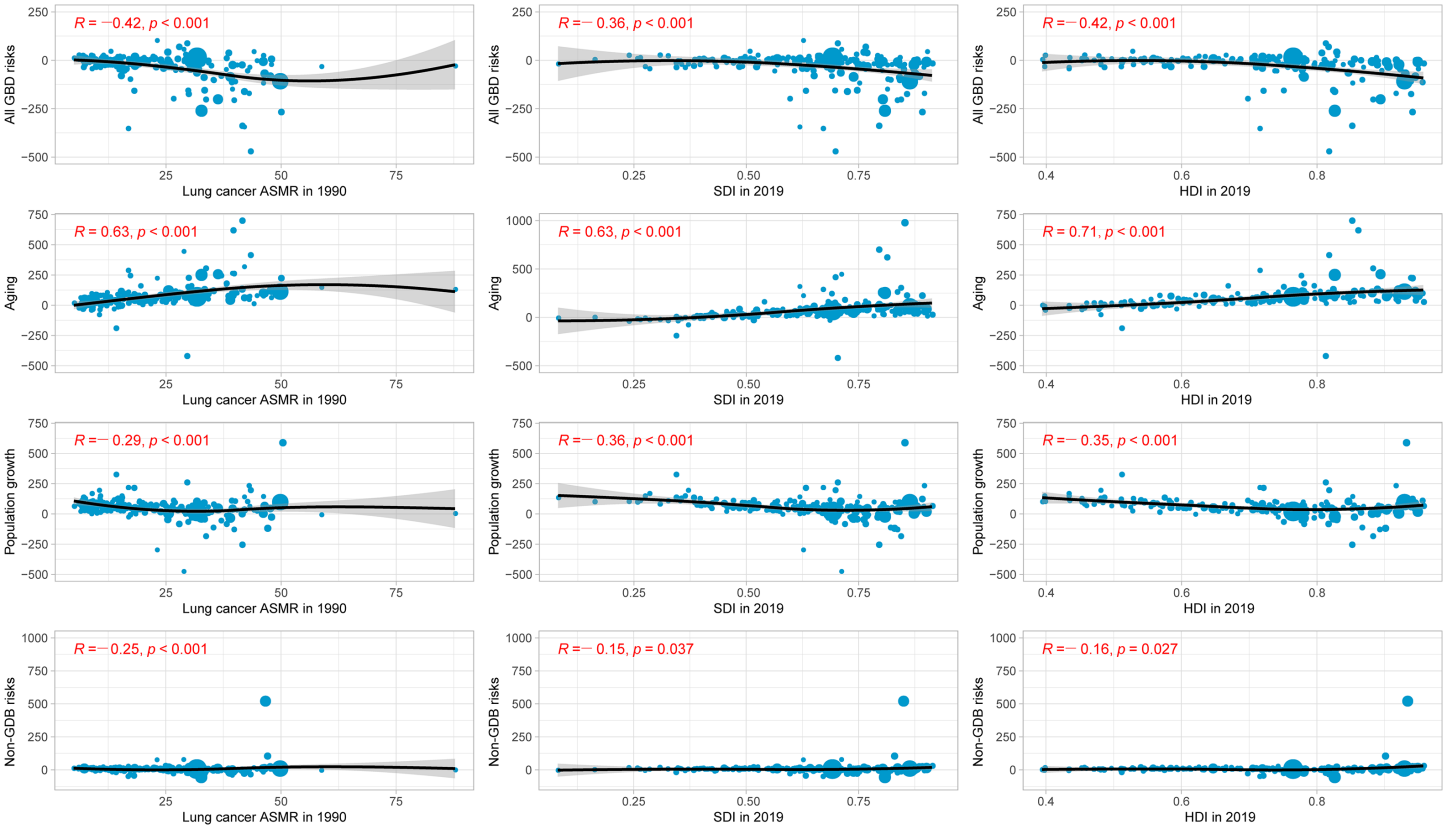
Percentage change in lung cancer mortality due to changes in population aging, population growth, and age-specific lung cancer mortality from all GBD risks and non-GBD risks, by lung cancer ASMR in 1990, SDI in 2019, and HDI in 2019.

We examined the relationship between ASMR in 1990, and SDI and HDI in 2019 and the contributions of all GBD risks, population growth, aging, and non-GBD risks (Figure 6) to the change in lung cancer deaths. We found that the contributions of all GBD risks, population growth, and non-GBD risks were all significantly and negatively associated with ASMR in 1990, and with SDI and HDI in 2019. In contrast, there was a significant positive association between the contribution of population aging and ASMR in 1990, and SDI and HDI in 2019. Furthermore, the contributions to age-specific lung cancer mortality from tobacco, air pollution, occupational risks, other environmental risks, and dietary risks were also significantly negatively associated with ASMR in 1990, and SDI and HDI in 2019 (Supplementary Figures S4–S6). However, the contribution of high fasting plasma glucose was significantly positively associated with ASMR in 1990, and insignificantly positively associated with the SDI and HDI in 2019.

## Discussion

This study provides a comprehensive and comparative assessment of the epidemiological and demographic drivers of lung cancer mortality by location and gender over the last three decades. Although a decline in ASMR was observed worldwide and in most regions, all-age mortality and the absolute number of lung cancer deaths continued to increase, mainly due to population aging and growth. The epidemiological and demographic drivers varied by region and sex and were negatively correlated with HDI, SDI in 2019 and lung cancer ASMR in 1990.

A decreasing trend in lung cancer burden in terms of incidence/mortality has been reported previously (7, 10, 24–26). Similar to these studies, we observed a decrease in lung cancer ASMR from all-cause or all GBD risks, which varied greatly by region and country. Non-GBD risk factors accounted for one-fifth of the global lung cancer deaths in 2019, and in this study, we observed a non-significant increase in ASMR for lung cancer from non-GBD risks, which has not been reported previously.

In cancer epidemiology, the effects of demographic factors are often removed in order to isolate modifiable risk factors. However, health system planning also requires an understanding of the absolute burden of disease and the impact of demographic factors. Currently, the combined effects of demographic and epidemiological drivers on lung cancer mortality are not well characterized at the global, regional, and national levels. A recent study found that the increase in global lung cancer cases from 1990 to 2019 was mainly attributable to population growth and aging (25). In this study, we observed similar contributions of population growth and aging to the absolute burden of lung cancer deaths. However, compared with the −7.92% decrease in the *per capita* burden of lung cancer incidence (25), a greater decrease in lung cancer deaths due to the combination of GBD and non-GBD risks was observed in our study. This may be due to advances in targeted treatments and immunotherapy, and to the increasing availability of screening and early detection through low-dose CT in recent years (27–29).

Most regions showed consistent upward or downward trends in lung cancer ASMR and changes in lung cancer deaths due to GBD risks and non-GBD risks. The notable geographical differences in the above two trends may be due to different levels of socio-economic development and changes in the spectrum of risk factors (30). First, and most importantly, effective tobacco control programs (awareness, education, and legal restrictions) have resulted in significant reductions in both lung cancer ASMR and absolute lung cancer deaths relative to age-specific mortality in some high SDI countries (31, 32). Second, with economic globalization, many occupational and environmental risk factors for lung cancer are shifting and becoming increasingly concentrated in lower-income countries. Therefore, exposure to occupational and environmental risks has been higher in low HDI regions (33, 34). Third, advances in early detection and treatment of lung cancer have opened the way to improved lung cancer survival in some high SDI countries, and even in some middle SDI countries, including China (28, 35). In contrast, lower SDI regions do not have the resources to support new treatments, as many are unable to provide widespread access to lung cancer screening and novel types of therapy (7). In general, countries with a higher baseline HDI provide better protective and preventive measures. This is supported by the negative correlation between the contributions to age-specific lung cancer mortality from all GBD risks, individual risk factors (except high fasting plasma glucose), and the SDI and HDI in 2019. Finally, countries with a low ASMR in 1990 are unlikely to have considered lung cancer a high priority in prevention programs because of its limited public health significance compared to other public health issues. Similar results were found for the ASMR for lung cancer (25, 26).

The diverse patterns of demographic and epidemiologic change in risk-specific lung cancer mortality around the world imply that tailored lung cancer control and prevention strategies are needed for individual regions, countries, and territories. Lung cancer is typically preventable because the risk factors are modifiable (36). Accordingly, primary prevention through risk factor mitigation, including the expansion of smoking control programs, and environmental measures to address air pollution, and occupational risks, must remain a priority in lung cancer control. This strategy is also appropriate for high and high-middle SDI regions, where the effectiveness of tobacco control has been demonstrated. In addition, these regions may still need to invest heavily in health promotion and treatment, given the trends in population aging and growth. For example, although the United Kingdom experienced the largest reduction in lung cancer deaths due to GBD risks, the absolute number of lung cancer deaths did not decline in 2019 compared with 1990. Similarly, the number of lung cancer deaths in the United States has increased by about 40% since 1990. In these regions, low-dose CT screening in high-risk populations and early targeted treatment can help further reduce lung cancer mortality.

Middle and low-middle SDI regions face a dual challenge from both demographic and epidemiological drivers (including all level 2 risks except dietary factors), with a typical example from East Asia. First, the smoking prevalence in middle and low-middle SDI countries has remained high in recent decades, suggesting much less progress in curbing smoking initiation or promoting cessation (19). Second, balancing the tradeoff between environmental/occupational protection and economic development is also a challenge for middle and low-middle SDI regions. Third, middle and low-middle SDI countries, similar to higher-income countries, face the challenges of population aging due to economic, infrastructural, and public health improvements (8). As a middle SDI country, China has the largest number of lung cancer deaths in the world, and lung cancer deaths are not only due to all GBD risk factors except dietary factors but also due to population aging and growth. The increase in lung cancer deaths in low SDI regions, including sub-Saharan Africa and Oceania, is mainly due to population growth. In these regions, more than 90% of the excess lung cancer deaths in 2019 were attributable to population growth.

In low, low-middle, and middle SDI regions, strategies to reduce lung cancer mortality in some high-income countries may not always be effective, feasible, or affordable. For example, the implementation of a screening program in low SDI regions may be premature because the limited downstream resources for treatment may be overwhelmed by the expectedly high number of cancer diagnoses in these regions. Instead, resources allocated to cancer control should focus on improvements in risk factor reduction. These include effective smoking control programs, stronger air quality regulations and enforcement, improved access to clean fuel sources, and legislation on the occupational risk factor. In addition, expanded cancer care and control strategies are needed to reduce disparities in lung cancer survival between developed and developing countries with limited resources (37–39).

The GBD risks in the decomposition analysis were further divided into six types of level 2 risks. The contributions to age-specific lung cancer mortality due to GBD risks were mainly from tobacco use, air pollution, occupational risks, other environmental risks, and dietary risks. However, increased lung cancer deaths due to high fasting plasma glucose were observed in most regions and were more pronounced in the regions with the greatest decrease in lung cancer deaths. This underscores the need for the continued worldwide promotion of a healthy lifestyle that includes physical exercise, the avoidance of obesity and hyperglycemia (40).

Lung cancer occurs more frequently in men than in women. While a decreasing trend in lung cancer ASMR in men has been observed in most regions, mainly due to effective tobacco control, an increasing trend has been observed in women worldwide and in most regions (7, 26, 41). Similarly, we found an increasing absolute burden of lung cancer deaths due to age-specific mortality from all GBD risks worldwide and in many regions. These differences between men and women also reflect differences in the stage and extent of the tobacco epidemic (42). In this study, regions such as Central and Western Europe had the highest burden of tobacco smoke-related lung cancer, which may be due to the later onset of smoking (43). In countries where the tobacco epidemic is at an earlier stage, like in China and several African countries, smoking has either recently peaked or continues to increase, and the burden of lung cancer in women due to tobacco smoking will continue to increase in the coming decades (44). In addition, the burden of lung cancer mortality from other level 2 risk factors has also increased in women worldwide because of economic globalization and associated social changes, such as the incorporation of women into the workforce.

The GBD 2019 used a comparative risk assessment approach to quantify associations between risk factors and outcomes. However, some common risk factors were not included in the risk-outcome associations for lung cancer, such as previous lung disease and genetic factors (45–47). In this study, we found a non-significant increase in lung cancer ASMR from non-GBD risks and an associated increase in absolute lung cancer deaths worldwide and in most regions/countries. Therefore, further studies are warranted to quantitatively assess the evidence and risk-outcome associations for non-GBD risks of lung cancer.

To our knowledge, this was the first study to systematically analyze the contributions of epidemiological and demographic drivers to lung cancer mortality at the global, regional, and country levels from 1990 to 2019. Our analysis broadens the perspective on the challenges that population aging, population growth, and epidemiological factors pose to the geographically and socioeconomically diverse lung cancer burden in most regions. However, as highlighted in previous GBD studies, our study has several limitations. First, the accuracy and robustness of GBD estimates are highly dependent on the quality and quantity of the data used for modeling. High-quality mortality data were lacking in some countries, especially in regions with lower SDIs (1). Second, the distribution of pathologic types of lung cancer has changed significantly in recent decades (37), but due to the lack of relevant data, temporal trends in lung cancer incidence by histology were not assessed in this study.

## Conclusion

In summary, we found that population aging and growth are responsible for the increase in the number of lung cancer deaths between 1990 and 2019, although age-specific death rates from GBD risks have decreased overall in most regions. The variation in the region- and country-specific demographic and epidemiologic changes in lung cancer mortality underscores the need for targeted strategies to reduce the burden of lung cancer.

## Data availability statement

Publicly available datasets were analyzed in this study. This data can be found at: http://ghdx.healthdata.org/gbd-results-tool.

## Ethics statement

Ethics approval and informed consent were not required for this study because of public accessibility to the data.

## Author contributions

YF, YJ, QZ, and YQ: conceptualization. LG, XL, JW, and HP: data curation. YF, HW, YW, and ZM: formal analysis. YF, ZS, and XL: writing—original draft. QZ and YQ: writing—review and editing. All authors contributed to the article and approved the submitted version.

## Funding

This work was supported by the China Medical Board (CMB, grant number: 21-445 to YQ), the Tianjin Natural Science Foundation (grant number: 22JCYBJC00290 to YF, 22JCYBJC00280 to XL). This work was also partly supported by the National Natural Science Foundation of China (grant number 81971650 to ZM, 82273019 to XL) and the Cancer Foundation of China (grant number: CFC2020KYXM001 and CFC2020KYXM002 to YF.

## Conflict of interest

The authors declare that the research was conducted in the absence of any commercial or financial relationships that could be construed as a potential conflict of interest.

## Publisher’s note

All claims expressed in this article are solely those of the authors and do not necessarily represent those of their affiliated organizations, or those of the publisher, the editors and the reviewers. Any product that may be evaluated in this article, or claim that may be made by its manufacturer, is not guaranteed or endorsed by the publisher.

## Abbreviations

GBD: Global burden of disease
EAPC: Estimated annual percentage changes
ASMR: Age-standardized mortality rate
CI: Confidence interval
UI: Uncertainty interval
SDI: Socio-demographic index
HDI: Human development index
CI5: Cancer incidence in five continents
SEER: Surveillance, epidemiology, and end results
NORDCAN: Nordic cancer registries database
