# Supplementary material for: Epidemiological and demographic drivers of lung cancer mortality from 1990 to 2019: results from the global burden of disease study 2019
Source: Front Public Health. 2023 May 5;11:1054200. doi: 10.3389/fpubh.2023.1054200 (PMC10196253; doi:10.3389/fpubh.2023.1054200)
Supplement: Supplementary file 1 [file Table_1.doc]

Table S1 Regional EAPC of all-age and Age-standardized lung cancer mortality from 1990 to 2019

| Location | All cause | | All GBD risks | | Non-GBD risks | |
| --- | --- | --- | --- | --- | --- | --- |
|  | All-age | ASMR | All-age | ASMR | All-age | ASMR |
| Global | 0.94(0.07-1.83) | -0.31(-1.1-0.49) | 0.78(-0.18-1.75) | -0.47(-1.34-0.41) | 1.7(-0.4-3.85) | 0.47(-1.44-2.42) |
| High SDI | 0.49(-0.08-1.06) | -0.91(-1.61--0.2) | 0.24(-0.38-0.86) | -1.15(-1.91--0.38) | 1.88(0.4-3.39) | 0.42(-1.37-2.24) |
| High-middle SDI | 0.94(0.25-1.63) | -0.5(-1.22-0.23) | 0.86(0.11-1.62) | -0.57(-1.36-0.22) | 1.31(-0.38-3.04) | -0.14(-1.9-1.65) |
| Middle SDI | 2.69(1.7-3.7) | 0.8(-0.03-1.65) | 2.66(1.55-3.79) | 0.75(-0.18-1.69) | 2.83(0.6-5.1) | 1.04(-0.86-2.97) |
| Low-middle SDI | 1.72(0.18-3.28) | 0.33(-0.84-1.51) | 1.52(-0.21-3.27) | 0.12(-1.19-1.44) | 2.49(-0.89-5.98) | 1.17(-1.45-3.85) |
| Low SDI | 0.14(-1.97-2.3) | 0.11(-1.25-1.49) | -0.02(-2.48-2.5) | -0.05(-1.62-1.55) | 0.6(-3.49-4.86) | 0.56(-2.12-3.31) |
| Andean Latin America | 1.18(-0.28-2.66) | -0.67(-1.84-0.51) | 0.75(-1.13-2.66) | -1.15(-2.65-0.37) | 1.82(-0.48-4.18) | 0.06(-1.8-1.96) |
| Australasia | 0.12(-0.54-0.79) | -1.25(-2.02--0.47) | -0.34(-1.07-0.39) | -1.7(-2.56--0.84) | 2.2(0.63-3.8) | 0.77(-1.06-2.63) |
| Caribbean | 1.51(0.56-2.48) | 0.03(-0.85-0.93) | 1.43(0.34-2.53) | -0.05(-1.06-0.96) | 1.8(-0.21-3.85) | 0.34(-1.53-2.24) |
| Central Asia | -1.27(-2.29--0.24) | -1.49(-2.35--0.62) | -1.35(-2.49--0.21) | -1.56(-2.51--0.59) | -0.94(-3.21-1.39) | -1.21(-3.14-0.75) |
| Central Europe | 1.5(0.96-2.05) | 0(-0.65-0.65) | 1.39(0.81-1.98) | -0.1(-0.81-0.6) | 2.17(0.7-3.66) | 0.62(-1.11-2.39) |
| Central Latin America | 1.18(-0.24-2.62) | -1.11(-2.23-0.02) | 0.66(-1.01-2.36) | -1.65(-2.96--0.31) | 2.48(-0.21-5.23) | 0.29(-1.85-2.49) |
| Central Sub-Saharan Africa | -0.85(-2.64-0.96) | -0.65(-1.76-0.46) | -1.15(-3.3-1.05) | -0.93(-2.25-0.42) | -0.21(-3.38-3.07) | -0.04(-2.03-1.98) |
| East Asia | 3.3(2.56-4.04) | 0.9(0.2-1.59) | 3.32(2.51-4.14) | 0.9(0.14-1.67) | 3.17(1.44-4.93) | 0.88(-0.76-2.54) |
| Eastern Europe | -1.12(-1.76--0.48) | -1.95(-2.7--1.19) | -1.17(-1.87--0.46) | -1.97(-2.81--1.13) | -0.92(-2.42-0.59) | -1.84(-3.6--0.05) |
| Eastern Sub-Saharan Africa | -0.05(-2.59-2.55) | 0.07(-1.44-1.61) | -0.28(-3.31-2.85) | -0.14(-1.94-1.69) | 0.46(-4.09-5.22) | 0.58(-2.19-3.43) |
| High-income Asia Pacific | 2.33(1.69-2.97) | -0.62(-1.43-0.2) | 2.09(1.39-2.8) | -0.8(-1.7-0.11) | 3.39(1.88-4.92) | 0.21(-1.7-2.16) |
| High-income North America | -0.15(-0.68-0.38) | -1.3(-1.93--0.68) | -0.4(-0.97-0.17) | -1.54(-2.21--0.86) | 1.29(-0.11-2.7) | 0.08(-1.56-1.74) |
| North Africa and Middle East | 1.52(0.14-2.92) | 0.34(-0.68-1.37) | 1.42(-0.09-2.96) | 0.25(-0.87-1.38) | 1.97(-1.32-5.37) | 0.8(-1.66-3.33) |
| Oceania | 0.79(-0.55-2.15) | 0.37(-0.52-1.26) | 0.69(-0.83-2.24) | 0.27(-0.73-1.29) | 1.13(-1.65-3.99) | 0.7(-1.2-2.64) |
| South Asia | 1.77(-0.12-3.71) | 0.11(-1.31-1.56) | 1.51(-0.63-3.69) | -0.17(-1.77-1.44) | 2.74(-1.34-6.99) | 1.24(-1.91-4.49) |
| Southeast Asia | 1.71(0.63-2.81) | 0.03(-0.84-0.9) | 1.5(0.26-2.75) | -0.19(-1.17-0.79) | 2.44(0.16-4.77) | 0.82(-1.04-2.71) |
| Southern Latin America | 0.13(-0.65-0.92) | -0.88(-1.68--0.08) | -0.04(-0.9-0.84) | -1.04(-1.92--0.14) | 0.82(-0.94-2.62) | -0.27(-2.06-1.56) |
| Southern Sub-Saharan Africa | 0.51(-0.64-1.68) | -0.42(-1.29-0.46) | 0.14(-1.14-1.44) | -0.78(-1.74-0.2) | 2.05(-0.59-4.77) | 1.18(-0.87-3.27) |
| Tropical Latin America | 1.67(0.52-2.82) | -0.43(-1.42-0.56) | 1.19(-0.08-2.47) | -0.9(-1.99-0.2) | 3.71(1.03-6.46) | 1.69(-0.66-4.09) |
| Western Europe | 0.47(-0.08-1.03) | -0.63(-1.35-0.09) | 0.27(-0.32-0.86) | -0.83(-1.6--0.05) | 1.8(0.27-3.36) | 0.63(-1.32-2.62) |
| Western Sub-Saharan Africa | 0.19(-1.99-2.41) | 0.82(-0.55-2.21) | -0.06(-2.73-2.69) | 0.59(-1.09-2.3) | 0.65(-3.04-4.47) | 1.27(-1.07-3.66) |

Table S2 Contribution of changes in population growth, population aging, and rates of age-specific lung cancer mortality to changes in lung cancer deaths by region, 1990–2019.

| Region | Population growth | Aging | Non-GBD risks | Tobacco | Air pollution | Occupational risks | High fasting plasma glucose | Other  environmental risks | Dietary risks |
| --- | --- | --- | --- | --- | --- | --- | --- | --- | --- |
| global | 56.73 | 59.58 | 3.5 | -12.66 | -3.47 | -3.52 | 1.83 | -0.78 | -1.2 |
| High_SDI | 55.64 | 99.32 | 7.98 | -40.93 | -9.48 | -11.6 | 2.88 | -1.82 | -1.98 |
| High_middle_SDI | 41.68 | 83.6 | -0.75 | -15.06 | -5.94 | -2.05 | 1.99 | -1.51 | -1.97 |
| Middle_SDI | 31.56 | 54.64 | 4.45 | 6.92 | -0.71 | 1.21 | 1.93 | 0.36 | -0.35 |
| Low_middle_SDI | 47.37 | 43.52 | 7.23 | -0.94 | -2.1 | 2.34 | 2.58 | 0.35 | -0.35 |
| Low_SDI | 92.21 | 4.25 | 5.09 | -2.84 | -2.75 | 1.34 | 2.29 | 0.2 | 0.22 |
| Andean Latin America | 62.45 | 64.29 | 0.70 | -11.13 | -10.84 | -5.9 | 2.15 | -0.51 | -1.2 |
| Australasia | 87.94 | 92.67 | 10.97 | -63.11 | -3.74 | -23.89 | 2.39 | -0.7 | -2.52 |
| Caribbean | 44.03 | 62.63 | 2.5 | -7 | -3.58 | 0.15 | 2.4 | -0.04 | -1.1 |
| Central Asia | 3103.25 | 1980.19 | -766.68 | -2647.67 | -980.6 | -350.36 | 82.63 | -277.45 | -243.31 |
| Central Europe | -22.13 | 118.48 | 7.62 | -4.89 | -12.61 | 7.32 | 7.34 | -0.33 | -0.81 |
| Central Latin America | 52.86 | 80.8 | 5.24 | -26.23 | -8.83 | -0.95 | -0.89 | -1.13 | -0.86 |
| Central Sub-Saharan Africa | 124.84 | -2.41 | 0.58 | -11.44 | -10.3 | -2.05 | 1.27 | -0.43 | -0.05 |
| East Asia | 17.46 | 66.66 | 2.94 | 10.02 | 0.16 | 1.28 | 1.55 | 0.46 | -0.53 |
| Eastern Europe | -34.9 | 120.21 | -31.7 | -92.14 | -35.85 | -8.78 | -0.38 | -8.07 | -8.4 |
| Eastern Sub-Saharan Africa | 98.01 | 0.02 | 5.77 | -3.69 | -3.35 | 1.81 | 1.5 | 0.09 | -0.14 |
| High-income Asia Pacific | 10.03 | 103.59 | 4.51 | -18.29 | -0.08 | 1.08 | 0.15 | 0.07 | -1.06 |
| High-income North America | 89.35 | 107.87 | 7.11 | -68.64 | -15.93 | -17.49 | 3.88 | -2.71 | -3.44 |
| North Africa and Middle East | 62.97 | 41.42 | 2.8 | -6.02 | -1.57 | -1.98 | 3.05 | -0.1 | -0.58 |
| Oceania | 74.33 | 15.32 | 5.55 | -0.98 | -0.1 | 1.75 | 3.75 | 0.22 | 0.18 |
| South Asia | 48.28 | 43.88 | 7.88 | -4.42 | -0.59 | 2.25 | 2.58 | 0.2 | -0.05 |
| Southeast Asia | 40.29 | 56.87 | 6.68 | -1.15 | -6.77 | 1.91 | 2.64 | 0.09 | -0.54 |
| Southern Latin America | 84.21 | 81.81 | -0.41 | -52.3 | -10.98 | -1.48 | 5 | -1.42 | -4.43 |
| Southern Sub-Saharan Africa | 64.5 | 51.81 | 10.24 | -18.75 | -6.49 | -3.84 | 3.23 | -0.3 | -0.4 |
| Tropical Latin America | 44.51 | 72.39 | 10.8 | -18.48 | -8.01 | -0.21 | 0.5 | -0.43 | -1.08 |
| Western Europe | 49.78 | 113.87 | 10.04 | -43.78 | -17.65 | -14.81 | 5.81 | -1.87 | -1.4 |
| Western Sub-Saharan Africa | 97.05 | -14.19 | 11.02 | 1.18 | 1.15 | -0.05 | 2.72 | 0.64 | 0.47 |

Table S3 Contribution of Changes in population growth, population aging, and rates of age-specific lung cancer mortality to changes in lung cancer deaths by country/territory, 1990–2019.

| location | Population growth | Aging | all GBD risks | Non-GBD risks | Percent change (%) |
| --- | --- | --- | --- | --- | --- |
| Afghanistan | 323.43 | -190.61 | -13.20 | -19.62 | 51.85 |
| Albania | -34.41 | 159.46 | -24.94 | -0.10 | 86.13 |
| Algeria | 60.89 | 65.22 | -23.19 | -2.92 | 131.82 |
| American Samoa | 19.72 | 90.79 | -13.78 | 3.28 | 103.68 |
| Andorra | 61.73 | 72.93 | -36.35 | 1.69 | 102.24 |
| Angola | 108.74 | -0.24 | -14.24 | 5.74 | 169.92 |
| Antigua and Barbuda | 67.39 | 42.70 | -4.85 | -5.23 | 75.56 |
| Argentina | 101.20 | 63.30 | -65.37 | 0.87 | 35.87 |
| Armenia | -56.61 | 243.84 | -82.51 | -4.72 | 24.75 |
| Australia | 89.49 | 89.05 | -89.41 | 10.87 | 53.09 |
| Austria | 55.99 | 97.44 | -47.30 | -6.14 | 28.01 |
| Azerbaijan | 69.15 | 70.14 | -35.45 | -3.84 | 64.19 |
| Bahamas | 50.98 | 73.59 | -22.04 | -2.52 | 115.79 |
| Bahrain | 143.24 | 92.78 | -118.24 | -17.77 | 128.13 |
| Bangladesh | 47.05 | 82.93 | -33.67 | 3.68 | 128.73 |
| Barbados | 32.03 | 71.31 | -12.01 | 8.67 | 65.17 |
| Belarus | -45.47 | 129.74 | -155.80 | -28.48 | -19.52 |
| Belgium | 587.16 | 976.41 | -1466.26 | 2.69 | 2.35 |
| Belize | 58.64 | 22.35 | 10.46 | 8.55 | 273 |
| Benin | 106.68 | -12.21 | -3.42 | 8.95 | 147.21 |
| Bermuda | 28.71 | 263.04 | -149.84 | -41.90 | 31.34 |
| Bhutan | 19.70 | 61.06 | 8.75 | 10.49 | 193.69 |
| Bolivia (Plurinational State of) | 59.13 | 40.13 | -8.76 | 9.50 | 185.61 |
| Bosnia and Herzegovina | -85.48 | 178.52 | 9.77 | -2.82 | 47.96 |
| Botswana | 57.36 | 29.11 | 4.86 | 8.67 | 174.57 |
| Brazil | 44.45 | 73.93 | -29.18 | 10.80 | 136.17 |
| Brunei Darussalam | 54.19 | 61.39 | -22.31 | 6.73 | 166.74 |
| Bulgaria | -84.65 | 100.24 | 68.46 | 15.96 | 31.02 |
| Burkina Faso | 97.32 | -17.77 | 8.47 | 11.98 | 143.59 |
| Burundi | 167.42 | -27.16 | -44.34 | 4.08 | 59.48 |
| Cabo Verde | 45.42 | 7.32 | 25.91 | 21.35 | 183.97 |
| Cambodia | 47.30 | 53.38 | -4.31 | 3.63 | 170.76 |
| Cameroon | 87.53 | -4.68 | 8.47 | 8.68 | 221.01 |
| Canada | 63.08 | 97.66 | -70.46 | 9.72 | 60.07 |
| Central African Republic | 147.16 | -9.31 | -33.34 | -4.51 | 57.33 |
| Chad | 101.96 | -41.17 | 24.94 | 14.27 | 173.43 |
| Chile | 41.74 | 78.22 | -22.17 | 2.20 | 115.07 |
| China | 17.39 | 66.57 | 13.18 | 2.85 | 195.39 |
| Colombia | 47.25 | 92.90 | -45.23 | 5.08 | 132.32 |
| Comoros | 58.14 | 48.71 | -12.37 | 5.52 | 108.6 |
| Congo | 105.81 | 19.45 | -23.43 | -1.83 | 107.69 |
| Cook Islands | -12.31 | 168.37 | -47.87 | -8.19 | 61.82 |
| Costa Rica | 48.87 | 72.82 | -28.21 | 6.51 | 150.53 |
| Croatia | -260.92 | 708.87 | -346.84 | -1.11 | 5.69 |
| Cuba | 7.72 | 91.64 | -3.82 | 4.46 | 87.59 |
| Cyprus | 49.48 | 34.11 | 13.65 | 2.76 | 185.57 |
| Czechia | 120.21 | 1299.87 | -1417.57 | -102.51 | -2.76 |
| Côte d’Ivoire | 72.59 | 22.83 | 3.30 | 1.27 | 181.22 |
| Democratic People's Republic of Korea | 30.31 | 65.74 | 6.24 | -2.29 | 107.71 |
| Democratic Republic of the Congo | 133.43 | -2.66 | -29.21 | -1.56 | 86.87 |
| Denmark | 121.38 | 222.35 | -268.32 | 24.59 | 10.53 |
| Djibouti | 54.42 | 35.67 | 7.03 | 2.88 | 407.45 |
| Dominica | -27.41 | 108.13 | -7.27 | 26.54 | 31.68 |
| Dominican Republic | 33.92 | 42.39 | 15.27 | 8.42 | 238.05 |
| Ecuador | 46.72 | 42.33 | 0.36 | 10.59 | 229.72 |
| Egypt | 51.61 | 19.80 | 22.69 | 5.91 | 200.84 |
| El Salvador | 20.26 | 62.62 | 1.21 | 15.91 | 135.98 |
| Equatorial Guinea | 115.76 | -32.01 | -0.82 | 17.06 | 186.33 |
| Eritrea | 67.18 | 18.24 | 4.37 | 10.21 | 223.47 |
| Estonia | -129.81 | 259.42 | -206.94 | -22.67 | -12.91 |
| Eswatini | 45.75 | 43.99 | -4.50 | 14.76 | 113.63 |
| Ethiopia | 134.23 | 3.68 | -36.87 | -1.04 | 74.13 |
| Fiji | 26.62 | 82.49 | -20.82 | 11.71 | 101.13 |
| Finland | 52.97 | 225.28 | -175.43 | -2.82 | 21.04 |
| France | 30.46 | 69.40 | -10.80 | 10.94 | 56.57 |
| Gabon | 93.25 | 8.59 | -7.58 | 5.75 | 83.87 |
| Gambia | 65.42 | 16.63 | 7.39 | 10.57 | 239.61 |
| Georgia | 257.53 | -419.23 | 86.06 | -24.36 | -6.35 |
| Germany | 16.27 | 82.28 | -9.92 | 11.38 | 45 |
| Ghana | 73.46 | 21.66 | -3.52 | 8.41 | 171.24 |
| Greece | -1.66 | 131.54 | -24.94 | -4.94 | 36.23 |
| Greenland | 1.95 | 130.05 | -30.30 | -1.71 | 79.7 |
| Grenada | 52.01 | 68.39 | -25.61 | 5.21 | 43.36 |
| Guam | 29.94 | 99.84 | -24.95 | -4.83 | 117.55 |
| Guatemala | 82.72 | 39.50 | -18.79 | -3.44 | 163.99 |
| Guinea | 107.26 | -34.91 | 22.20 | 5.45 | 95.94 |
| Guinea-Bissau | 131.25 | -16.11 | -21.56 | 6.42 | 62.9 |
| Guyana | 0.16 | 95.76 | -7.34 | 11.42 | 70.29 |
| Haiti | 120.50 | 18.56 | -33.64 | -5.42 | 75.13 |
| Honduras | 45.90 | 23.14 | 18.34 | 12.62 | 379.13 |
| Hungary | -28.20 | 113.34 | 4.80 | 10.05 | 29.16 |
| Iceland | 65.16 | 73.74 | -44.92 | 6.02 | 60.51 |
| India | 43.80 | 44.07 | 4.25 | 7.88 | 201.8 |
| Indonesia | 31.60 | 44.25 | 16.14 | 8.01 | 190.8 |
| Iran (Islamic Republic of) | 32.74 | 62.06 | 0.54 | 4.66 | 208.89 |
| Iraq | 70.90 | 17.45 | 6.31 | 5.34 | 235.78 |
| Ireland | 108.12 | 97.52 | -114.68 | 9.04 | 33.68 |
| Israel | 80.59 | 32.08 | -17.02 | 4.35 | 117.84 |
| Italy | 42.89 | 254.04 | -203.21 | 6.28 | 15.09 |
| Jamaica | 23.83 | 46.78 | 17.01 | 12.38 | 108.17 |
| Japan | 2.24 | 117.27 | -24.48 | 4.98 | 102.55 |
| Jordan | 64.50 | 30.50 | 2.35 | 2.65 | 443.03 |
| Kazakhstan | 31.08 | 59.34 | -162.51 | -27.91 | -32.7 |
| Kenya | 62.33 | 16.27 | 10.84 | 10.56 | 236.88 |
| Kiribati | 80.95 | 28.17 | -7.72 | -1.39 | 78.79 |
| Kuwait | 77.91 | 48.92 | -21.49 | -5.34 | 230.87 |
| Kyrgyzstan | 109.57 | 19.01 | -199.18 | -29.40 | -31.65 |
| Lao People's Democratic Republic | 93.06 | 34.55 | -31.21 | 3.60 | 79.91 |
| Latvia | -113.87 | 123.74 | -101.52 | -8.36 | -25.23 |
| Lebanon | 42.54 | 37.36 | 19.43 | 0.66 | 189.61 |
| Lesotho | 23.04 | 16.20 | 45.94 | 14.83 | 89.43 |
| Liberia | 193.95 | -79.08 | -19.75 | 4.87 | 61.31 |
| Libya | 50.69 | 58.92 | -11.36 | 1.76 | 151.23 |
| Lithuania | -185.52 | 303.33 | -203.32 | -14.49 | -13.97 |
| Luxembourg | 232.34 | 47.42 | -172.14 | -7.61 | 23.72 |
| Madagascar | 126.27 | -11.49 | -22.58 | 7.80 | 90.52 |
| Malawi | 92.38 | -3.17 | 4.37 | 6.43 | 103.41 |
| Malaysia | 47.21 | 43.71 | 1.49 | 7.59 | 234.31 |
| Maldives | 95.55 | 62.74 | -56.26 | -2.03 | 138.83 |
| Mali | 109.34 | -25.62 | 12.76 | 3.51 | 135.42 |
| Malta | 34.08 | 117.94 | -52.92 | 0.89 | 66.71 |
| Marshall Islands | 28.13 | 62.94 | 2.49 | 6.44 | 118.99 |
| Mauritania | 93.65 | 9.20 | -6.33 | 3.47 | 102.91 |
| Mauritius | 23.58 | 114.83 | -35.23 | -3.18 | 93.36 |
| Mexico | 66.36 | 114.13 | -80.73 | 0.24 | 81.99 |
| Micronesia (Federated States of) | -4.30 | 84.16 | 10.51 | 9.63 | 64.41 |
| Monaco | 29.82 | 11.26 | 44.04 | 14.88 | 103.71 |
| Mongolia | 101.51 | 65.06 | -55.00 | -11.57 | 57.3 |
| Montenegro | -1.47 | 76.23 | 23.60 | 1.64 | 82.19 |
| Morocco | 36.77 | 50.97 | 4.95 | 7.31 | 160.91 |
| Mozambique | 80.60 | -23.75 | 26.18 | 16.97 | 176.35 |
| Myanmar | 50.18 | 74.02 | -34.19 | 9.99 | 77.45 |
| Namibia | 63.19 | 14.07 | 8.24 | 14.49 | 130.73 |
| Nauru | 140.00 | 318.10 | -345.97 | -12.13 | 2.06 |
| Nepal | 44.60 | 47.14 | 2.29 | 5.97 | 168.5 |
| Netherlands | 43.19 | 119.59 | -83.18 | 20.40 | 38.65 |
| New Zealand | 77.35 | 114.45 | -103.48 | 11.68 | 43.46 |
| Nicaragua | 45.05 | 48.49 | -3.37 | 9.83 | 214.01 |
| Niger | 99.33 | -2.14 | 2.80 | 0.01 | 192.05 |
| Nigeria | 110.56 | -30.34 | 2.76 | 17.03 | 121.42 |
| Niue | -477.22 | 444.09 | 56.09 | 77.03 | 7.34 |
| North Macedonia | 8.24 | 57.40 | 30.23 | 4.12 | 125.75 |
| Northern Mariana Islands | -8.54 | 146.31 | -33.27 | -4.50 | 141.44 |
| Norway | 63.69 | 25.18 | -17.48 | 28.61 | 43.7 |
| Oman | 97.96 | 13.63 | -18.78 | 7.18 | 141.63 |
| Pakistan | 83.92 | -3.98 | 8.98 | 11.07 | 125.4 |
| Palau | 20.83 | 78.24 | -2.66 | 3.59 | 115.38 |
| Palestine | 80.02 | 11.00 | 5.15 | 3.84 | 193.85 |
| Panama | 77.22 | 67.87 | -49.58 | 4.48 | 107.56 |
| Papua New Guinea | 81.44 | 4.73 | 8.32 | 5.51 | 191.57 |
| Paraguay | 41.04 | 30.04 | 18.73 | 10.19 | 266.53 |
| Peru | 72.28 | 91.44 | -54.30 | -9.41 | 89 |
| Philippines | 83.51 | 59.01 | -42.06 | -0.46 | 100.4 |
| Poland | 1.69 | 99.66 | -7.84 | 6.50 | 54.07 |
| Portugal | 9.78 | 84.50 | -0.87 | 6.59 | 66.93 |
| Puerto Rico | -8.87 | 243.61 | -98.52 | -36.22 | 35.84 |
| Qatar | 95.93 | 20.02 | -14.23 | -1.72 | 615.79 |
| Republic of Korea | 16.12 | 80.65 | 0.20 | 3.03 | 230.48 |
| Republic of Moldova | -67.67 | 150.98 | -157.08 | -26.23 | -24.79 |
| Romania | -48.65 | 88.35 | 41.19 | 19.11 | 50.48 |
| Russian Federation | -26.06 | 247.24 | -262.86 | -58.33 | -10.63 |
| Rwanda | 95.42 | 27.41 | -12.79 | -10.04 | 82.13 |
| Saint Kitts and Nevis | 98.58 | 45.72 | -26.43 | -17.87 | 45.18 |
| Saint Lucia | 32.32 | 88.46 | -20.52 | -0.26 | 114.54 |
| Saint Vincent and the Grenadines | 4.12 | 87.94 | 0.23 | 7.71 | 99.23 |
| Samoa | 53.24 | 51.93 | -6.97 | 1.80 | 61.67 |
| San Marino | 56.77 | 49.56 | -14.29 | 7.96 | 81.73 |
| Sao Tome and Principe | 70.36 | -11.60 | 20.81 | 20.44 | 110.63 |
| Saudi Arabia | 70.44 | 27.33 | 1.54 | 0.69 | 207.17 |
| Senegal | 71.82 | 16.95 | 3.05 | 8.18 | 157.07 |
| Serbia | -15.92 | 73.92 | 37.12 | 4.88 | 57.81 |
| Seychelles | 60.63 | 54.34 | -13.74 | -1.23 | 74.53 |
| Sierra Leone | 126.59 | -37.30 | -2.97 | 13.68 | 92.96 |
| Singapore | 104.32 | 119.67 | -110.76 | -13.23 | 90.08 |
| Slovakia | 44.73 | 618.65 | -559.68 | -3.71 | 6.8 |
| Slovenia | 12.96 | 116.40 | -28.95 | -0.42 | 48.86 |
| Solomon Islands | 71.62 | 18.03 | 6.43 | 3.92 | 147.13 |
| Somalia | 132.97 | -8.90 | -19.48 | -4.60 | 123.69 |
| South Africa | 67.48 | 59.62 | -35.98 | 8.88 | 84.73 |
| South Sudan | 147.69 | -3.79 | -38.13 | -5.77 | 36.96 |
| Spain | 44.39 | 85.56 | -33.20 | 3.25 | 47.26 |
| Sri Lanka | 22.95 | 60.50 | 5.30 | 11.25 | 187.79 |
| Sudan | 95.22 | -2.64 | -0.26 | 7.68 | 108.95 |
| Suriname | 41.38 | 46.60 | 7.05 | 4.97 | 161.75 |
| Sweden | 49.58 | 38.06 | -0.62 | 12.99 | 42.02 |
| Switzerland | 1765.25 | 1734.12 | -3221.68 | -177.69 | 1.44 |
| Syrian Arab Republic | 13.24 | 80.01 | 1.55 | 5.20 | 149.77 |
| Taiwan (Province of China) | 13.58 | 72.53 | 2.57 | 11.32 | 204.11 |
| Tajikistan | 1364.61 | 160.07 | -1305.39 | -119.29 | 4.61 |
| Thailand | 28.24 | 115.06 | -45.27 | 1.97 | 118.86 |
| Timor-Leste | 40.92 | 46.42 | 6.98 | 5.68 | 266.58 |
| Togo | 65.80 | 27.04 | 0.85 | 6.31 | 217.11 |
| Tokelau | -298.42 | 222.17 | 101.41 | 74.83 | 6.23 |
| Tonga | 13.71 | 80.37 | -7.40 | 13.32 | 50.74 |
| Trinidad and Tobago | 23.06 | 103.94 | -27.41 | 0.41 | 89.55 |
| Tunisia | 34.59 | 66.52 | -3.19 | 2.08 | 152.4 |
| Turkey | 42.19 | 81.65 | -25.59 | 1.75 | 110.57 |
| Turkmenistan | 216.90 | 287.71 | -354.04 | -50.57 | 16.57 |
| Tuvalu | 50.51 | 40.64 | -4.64 | 13.48 | 58.28 |
| Uganda | 90.00 | -11.73 | 10.16 | 11.57 | 160.57 |
| Ukraine | -36.25 | 41.01 | -87.19 | -17.57 | -39.53 |
| United Arab Emirates | 72.40 | 30.96 | -2.84 | -0.52 | 738.57 |
| United Kingdom | 5371.41 | 5243.95 | -11026.58 | 511.22 | 0.29 |
| United Republic of Tanzania | 89.50 | 1.67 | 3.21 | 5.62 | 138.97 |
| United States of America | 94.08 | 109.46 | -110.21 | 6.67 | 31.96 |
| United States Virgin Islands | -1.89 | 76.42 | 17.89 | 7.58 | 196.47 |
| Uruguay | 194.73 | 413.13 | -471.97 | -35.90 | 4.78 |
| Uzbekistan | 213.71 | 90.66 | -158.64 | -45.73 | 26.3 |
| Vanuatu | 57.17 | 28.38 | 7.68 | 6.76 | 214.77 |
| Venezuela (Bolivarian Republic of) | 35.99 | 63.62 | -7.59 | 7.98 | 209.41 |
| Viet Nam | 34.15 | 48.11 | 8.75 | 9.00 | 178.99 |
| Yemen | 79.24 | 17.60 | -0.78 | 3.94 | 181.36 |
| Zambia | 91.13 | 1.90 | 2.66 | 4.31 | 147.75 |
| Zimbabwe | 60.76 | 25.58 | -3.88 | 17.54 | 84.08 |

Table S4 Contribution of specific GBD risks to changes in lung cancer deaths by country/territory, 1990–2019.

| Country/ territory | Tobacco | Air pollution | Occupational risks | High fasting plasma glucose | Other environmental risks | dietary risks |
| --- | --- | --- | --- | --- | --- | --- |
| Afghanistan | 8.37 | -21.88 | -3.22 | 6.32 | -1.4 | -1.78 |
| Albania | -7.67 | -15.48 | 1.16 | 2.13 | -1.11 | -3.91 |
| Algeria | -17.55 | -4.01 | -1.06 | 2.67 | -0.65 | -2.61 |
| American Samoa | -9.07 | -7.14 | -2.34 | 4.84 | -0.15 | 0.04 |
| Andorra | -21.42 | -5.81 | -9.12 | 1.64 | -1.09 | -0.68 |
| Angola | -3.52 | -9.28 | -1.91 | 1.41 | -0.06 | -0.95 |
| Antigua and Barbuda | -3.31 | -2.05 | -3.93 | 4.72 | -0.24 | -0.05 |
| Argentina | -53.31 | -13.21 | 2.69 | 5.25 | -1.38 | -5.37 |
| Armenia | -58.48 | -17.61 | 0.2 | 7.88 | -6.96 | -7.34 |
| Australia | -61.64 | -3.7 | -23.34 | 2.38 | -0.5 | -2.6 |
| Austria | -22.65 | -21.02 | -6.06 | 8.56 | -2.91 | -3.18 |
| Azerbaijan | -21.85 | -12.36 | -1.32 | 4.2 | -1.59 | -2.48 |
| Bahamas | -14.9 | -3.58 | -3.59 | 1.58 | -0.46 | -1.06 |
| Bahrain | -71.05 | -27.18 | -7.26 | -8.11 | -2.69 | -1.84 |
| Bangladesh | -20.91 | -9.34 | -2.13 | 0.79 | -0.58 | -1.4 |
| Barbados | -14.75 | -0.34 | -0.68 | 4.68 | -0.06 | -0.86 |
| Belarus | -97.7 | -39.28 | -5.96 | -1.61 | -2.23 | -9.13 |
| Belgium | -807.05 | -254.84 | -324.27 | 24.46 | -45.4 | -57.65 |
| Belize | 3.88 | -0.09 | 3.33 | 2.84 | 0.39 | 0.12 |
| Benin | -5.76 | -0.72 | -0.12 | 2.78 | 0.29 | 0.13 |
| Bermuda | -67.11 | -21.37 | -54.52 | -3.09 | -3.82 | 0.18 |
| Bhutan | 2.15 | -0.72 | 2.83 | 3.15 | 0.95 | 0.38 |
| Bolivia (Plurinational State of) | -9.39 | -5.25 | 3.18 | 2.32 | 0.1 | 0.24 |
| Bosnia and Herzegovina | 13.93 | -11.82 | -1.68 | 11.79 | 0.39 | -2.83 |
| Botswana | 3.34 | -2.43 | -0.41 | 3.15 | 0.49 | 0.71 |
| Brazil | -19.4 | -8.21 | -0.36 | 0.4 | -0.46 | -1.14 |
| Brunei Darussalam | -16.87 | -2.47 | -2.02 | -0.71 | -0.09 | -0.17 |
| Bulgaria | 44.93 | -3.82 | 7.34 | 12.18 | 2.44 | 5.38 |
| Burkina Faso | 0.98 | 3.05 | -0.22 | 2.86 | 0.71 | 1.09 |
| Burundi | -28.74 | -11.28 | -2.79 | 1.14 | -0.83 | -1.68 |
| Cabo Verde | 8.09 | 6.8 | 1.64 | 6.2 | 1.71 | 1.5 |
| Cambodia | -4.99 | -4.76 | 1.94 | 3.65 | -0.02 | -0.16 |
| Cameroon | 1.18 | 2.51 | 0.89 | 2.6 | 0.62 | 0.69 |
| Canada | -50.42 | -6.52 | -13.4 | 2.61 | -1.1 | -1.64 |
| Central African Republic | -17.16 | -12.59 | -3.75 | 1.92 | -0.85 | -0.87 |
| Chad | 9.11 | 7.86 | 1.43 | 3.57 | 1.14 | 1.83 |
| Chile | -16.03 | -3.99 | -3.71 | 3.31 | -0.42 | -1.32 |
| China | 10.25 | 0.2 | 1.26 | 1.53 | 0.47 | -0.54 |
| Colombia | -28.58 | -11.2 | -1.59 | -1.16 | -1.25 | -1.41 |
| Comoros | -8.6 | -6.89 | 1.49 | 1.1 | -0.12 | 0.68 |
| Congo | -8.14 | -11.61 | -3.38 | 1.05 | -0.48 | -0.93 |
| Cook Islands | -37.77 | -7.13 | -2.54 | 3.94 | -1.53 | -2.84 |
| Costa Rica | -20.33 | -6.3 | -1.28 | 0.84 | -0.6 | -0.51 |
| Croatia | -325.41 | -114.89 | 125.23 | 13.91 | -16.98 | -21.51 |
| Cuba | -3.98 | -2.24 | 0.66 | 2.86 | 0.01 | -1.15 |
| Cyprus | 10.24 | 0.74 | -0.77 | 2.37 | 0.08 | 0.96 |
| Czechia | 890.58 | 283.49 | 143.19 | -37.39 | 101.24 | 40.83 |
| Côte d’Ivoire | 2.52 | -1.3 | -0.36 | 1.94 | 0.13 | 0.37 |
| Democratic People's Republic of Korea | 4.21 | -2.34 | 2.11 | 2.06 | 0.07 | 0.14 |
| Democratic Republic of the Congo | -17.33 | -10.63 | -2.11 | 1.02 | -0.7 | 0.43 |
| Denmark | -171.42 | -53.31 | -33.73 | 17.35 | -8.48 | -18.73 |
| Djibouti | 4.69 | -0.56 | 1.13 | 1.23 | 0.24 | 0.35 |
| Dominica | -15.49 | -17.51 | 8.61 | 16.67 | 0.51 | 0.03 |
| Dominican Republic | 11.55 | -2.29 | 2.82 | 2.89 | 0.53 | -0.25 |
| Ecuador | -4.2 | -2.09 | 2.28 | 3.52 | 0.4 | 0.48 |
| Egypt | 11.19 | 5.23 | 2.5 | 4.33 | 0.19 | -0.76 |
| El Salvador | -0.6 | -7.08 | 2.21 | 5.62 | 0.73 | 0.32 |
| Equatorial Guinea | 0.17 | -4.73 | 0.84 | 3.24 | 0.59 | -1.14 |
| Eritrea | -1.12 | 0.96 | 1.97 | 1.65 | 0.45 | 0.48 |
| Estonia | -135.56 | -47.37 | -6.47 | 4.77 | -11.54 | -11.04 |
| Eswatini | -6.16 | -2.74 | -0.16 | 3.72 | 0.57 | 0.33 |
| Ethiopia | -16.27 | -16.74 | -0.51 | -0.92 | -0.94 | -1.64 |
| Fiji | -18.21 | -10.46 | 2.25 | 6.27 | -0.14 | -0.55 |
| Finland | -110.3 | -15.95 | -36.84 | 2.63 | -8.75 | -6.01 |
| France | -9.13 | -7.32 | 2.19 | 2.93 | 0.21 | 0.33 |
| Gabon | -1.07 | -9.95 | -1.25 | 3.69 | 0.02 | 0.94 |
| Gambia | -0.29 | 2.89 | 0.92 | 2.3 | 0.64 | 0.92 |
| Georgia | -11.5 | -53.58 | 91.93 | 57.35 | -2.82 | 5.63 |
| Germany | -8.01 | -10.19 | -0.5 | 7.34 | 0.28 | 1.2 |
| Ghana | -1.97 | -0.86 | -2.63 | 3.03 | 0.36 | -1.48 |
| Greece | -13.54 | -12.28 | -4.71 | 6.34 | -1.48 | 0.76 |
| Greenland | -29.6 | -3.27 | 3.14 | 3.42 | -2.26 | -1.72 |
| Grenada | -9.8 | -11.87 | -13.47 | 9.46 | -0.2 | 0.15 |
| Guam | -17.49 | -2.95 | -4.02 | 0.94 | -0.63 | -0.78 |
| Guatemala | -9.29 | -10.12 | -0.44 | 2.93 | -0.79 | -1.06 |
| Guinea | 12.1 | 2.59 | 1.85 | 3.54 | 0.76 | 1.34 |
| Guinea-Bissau | -13.35 | -8.06 | -2.66 | 3.11 | -0.26 | -0.4 |
| Guyana | -9.71 | -4.59 | -0.02 | 6.51 | 0.13 | 0.36 |
| Haiti | -21.51 | -13.15 | -1 | 2.61 | -0.72 | 0.2 |
| Honduras | 5.2 | 3.36 | 4.12 | 3.7 | 1.03 | 0.88 |
| Hungary | 2.66 | -16.85 | 5.41 | 11.05 | 0.89 | 1.67 |
| Iceland | -38.08 | -3.57 | -4.23 | 3.98 | -0.35 | -2.64 |
| India | -1.8 | 0.64 | 2.33 | 2.77 | 0.29 | 0 |
| Indonesia | 12.38 | -2.75 | 3.1 | 2.87 | 0.39 | 0.14 |
| Iran (Islamic Republic of) | -3.02 | 0.07 | 0.43 | 3.19 | 0.16 | -0.29 |
| Iraq | 1.3 | 0.53 | 0.15 | 3.16 | 0.29 | 0.87 |
| Ireland | -74.19 | -15.24 | -24.25 | 10.06 | -4.75 | -6.24 |
| Israel | -14.89 | -2.72 | -1.86 | 2.54 | -0.41 | 0.32 |
| Italy | -115.76 | -43.82 | -40.19 | 5.96 | -6.98 | -2.34 |
| Jamaica | 10.48 | -5.66 | 2.73 | 7.69 | 0.66 | 1.13 |
| Japan | -23.47 | -1.24 | 2.29 | -0.56 | -0.29 | -1.21 |
| Jordan | 0 | 0.93 | -0.85 | 1.89 | 0.14 | 0.23 |
| Kazakhstan | -93.76 | -31.35 | -17.08 | -0.74 | -10.49 | -9.12 |
| Kenya | 2.55 | 1.85 | 3.51 | 1.68 | 0.63 | 0.6 |
| Kiribati | -3.84 | -11.03 | 1.19 | 6.71 | -0.2 | -0.59 |
| Kuwait | -16.21 | -6.18 | 1.48 | 0.42 | -0.47 | -0.49 |
| Kyrgyzstan | -118.31 | -45.67 | -11.38 | -2.43 | -11.98 | -9.46 |
| Lao People's Democratic Republic | -20.99 | -11.3 | 0.81 | 3.45 | -0.37 | -2.93 |
| Latvia | -64.88 | -27.44 | -2.25 | 3.03 | -6.26 | -3.72 |
| Lebanon | 10.66 | 2.47 | 0.45 | 4.67 | 0.51 | 0.64 |
| Lesotho | 17.93 | 6.69 | 12.3 | 4.86 | 1.88 | 2.26 |
| Liberia | -14.55 | -7.71 | -1.36 | 4.04 | -0.39 | 0.18 |
| Libya | -10.44 | -3.27 | -1.22 | 3.72 | -0.28 | 0.12 |
| Lithuania | -120.19 | -49.05 | -22.05 | 1.95 | -6.34 | -7.74 |
| Luxembourg | -106.34 | -25.99 | -43.18 | 18.45 | -10.08 | -4.62 |
| Madagascar | -19.49 | -5.14 | 0.93 | 1.4 | -0.29 | 0.05 |
| Malawi | 0.82 | -1.17 | 1.99 | 2.52 | 0.28 | -0.09 |
| Malaysia | 0.39 | -2.98 | 1.74 | 2.85 | 0.12 | -0.62 |
| Maldives | -26.12 | -20.65 | -6.66 | 0.48 | -1.32 | -1.99 |
| Mali | 9.1 | 0.18 | 0.32 | 2.6 | 0.4 | 0.17 |
| Malta | -33.82 | -6.78 | -10.01 | 0.39 | -1.18 | -1.46 |
| Marshall Islands | 0.07 | -5.01 | 0.11 | 6.79 | 0.28 | 0.27 |
| Mauritania | -2.02 | -4.32 | -1.71 | 1.87 | -0.03 | -0.04 |
| Mauritius | -28.29 | -5.32 | -3.29 | 4.9 | -0.74 | -2.52 |
| Mexico | -49.8 | -17.77 | -3.14 | -5.25 | -2.79 | -1.97 |
| Micronesia (Federated States of) | 7.24 | -13.28 | 4.29 | 11.21 | 0.49 | 0.63 |
| Monaco | 22.75 | 4.36 | 8.75 | 6.28 | 1.85 | 0.06 |
| Mongolia | -25 | -19.88 | -3.87 | 0.92 | -4.06 | -3.08 |
| Montenegro | 18.82 | -3.69 | 2.39 | 6.32 | 0.83 | -1.06 |
| Morocco | -0.98 | 1.89 | 0.83 | 4.46 | 0.5 | -1.71 |
| Mozambique | 8.06 | 7.82 | 4.22 | 3.27 | 1.06 | 1.69 |
| Myanmar | -26.85 | -11.63 | 2.9 | 3.31 | -0.32 | -1.66 |
| Namibia | 1.23 | -0.61 | 2.88 | 2.86 | 0.78 | 1.09 |
| Nauru | -281.09 | -216.51 | 3.53 | 155.4 | -7.75 | 1.52 |
| Nepal | -2.31 | -0.94 | 2.57 | 2.83 | 0.32 | -0.21 |
| Netherlands | -42.86 | -13.4 | -26.38 | 1.57 | -0.42 | -1.66 |
| New Zealand | -70.84 | -4.02 | -27.13 | 2.35 | -1.72 | -2.14 |
| Nicaragua | -5.58 | -3.51 | 2.23 | 2.57 | 0.29 | 0.66 |
| Niger | 3.42 | -2.04 | -0.21 | 1.94 | 0.03 | -0.34 |
| Nigeria | -1.22 | 0.48 | -0.56 | 2.49 | 0.84 | 0.71 |
| Niue | 18.65 | -68.52 | 15.49 | 86.44 | 3.31 | 0.78 |
| North Macedonia | 19.66 | 0.36 | 1.3 | 6.64 | 2.11 | 0.18 |
| Northern Mariana Islands | -25.24 | -6.76 | -1.55 | 1.76 | -0.86 | -0.63 |
| Norway | -19.88 | -8.6 | 4.87 | 6.47 | 0.94 | -1.28 |
| Oman | -17.15 | -4.21 | -0.05 | 3.67 | -0.22 | -0.79 |
| Pakistan | -0.99 | 0.79 | 4.28 | 3.51 | 0.63 | 0.77 |
| Palau | -8.36 | -0.2 | 0.34 | 5.97 | -0.08 | -0.36 |
| Palestine | 1 | 0.03 | -0.57 | 4.54 | 0.27 | -0.12 |
| Panama | -34.92 | -12.33 | -0.49 | 0.96 | -1.33 | -1.45 |
| Papua New Guinea | 1.51 | 0.92 | 1.94 | 3.39 | 0.29 | 0.29 |
| Paraguay | 11.06 | -1.54 | 3.81 | 3.73 | 0.7 | 0.98 |
| Peru | -16.96 | -18.74 | -15.52 | 1.17 | -1.39 | -2.87 |
| Philippines | -23.36 | -15.06 | -1.86 | -0.16 | -0.54 | -1.05 |
| Poland | -11.1 | -11.22 | 11.02 | 4.75 | -0.15 | -1.14 |
| Portugal | -6.37 | -5.34 | 5.48 | 5.84 | 0.09 | -0.56 |
| Puerto Rico | -64.83 | -5.95 | -13.65 | -3.86 | -2.84 | -7.25 |
| Qatar | -10.33 | -3.69 | -1.05 | 1.4 | -0.32 | -0.24 |
| Republic of Korea | -2.92 | 1.09 | 0.55 | 1.91 | 0.04 | -0.46 |
| Republic of Moldova | -84.3 | -53.8 | -6.4 | -1.46 | -6.88 | -4.22 |
| Romania | 32.14 | -5.65 | 3.59 | 7.86 | 2.3 | 0.97 |
| Russian Federation | -153.77 | -61.65 | -16.92 | 1.3 | -15.51 | -16.36 |
| Rwanda | -2.3 | -10.07 | -0.15 | 0.66 | -0.76 | -0.03 |
| Saint Kitts and Nevis | -9 | -8.06 | -8.87 | 3.04 | -0.9 | -2.56 |
| Saint Lucia | -11.72 | -8.12 | -2.99 | 1.22 | -0.36 | 1.46 |
| Saint Vincent and the Grenadines | -0.25 | -3.91 | 1.31 | 4.73 | 0.21 | -1.86 |
| Samoa | -3.9 | -7.12 | -1.81 | 6.37 | -0.08 | -0.5 |
| San Marino | -14.51 | -2.89 | 0.15 | 3.67 | -0.23 | -0.27 |
| Sao Tome and Principe | 12.12 | 2.14 | 1.27 | 4.69 | 1.62 | -1.07 |
| Saudi Arabia | -1.07 | -0.1 | -0.84 | 2.85 | 0 | 0.69 |
| Senegal | -1.91 | 1.05 | 0.23 | 2.89 | 0.41 | 0.37 |
| Serbia | 26.49 | -4.61 | 5.21 | 9.01 | 1.9 | -0.85 |
| Seychelles | -15.45 | -2.94 | -2.26 | 8.79 | -0.31 | -1.59 |
| Sierra Leone | -7.19 | 0.94 | -0.44 | 2.61 | 0.55 | 0.56 |
| Singapore | -65.79 | -13.98 | -18.73 | -7.22 | -0.76 | -4.26 |
| Slovakia | -354.18 | -111.5 | -42.61 | 0.48 | -30.43 | -20.16 |
| Slovenia | -22.1 | -14.83 | 9.46 | 1.7 | -1.63 | -1.36 |
| Solomon Islands | -0.68 | -0.79 | 3.05 | 4.66 | 0.17 | -0.01 |
| Somalia | -9.97 | -8.59 | -0.35 | 0.69 | -0.52 | -0.71 |
| South Africa | -23.85 | -8.08 | -5.61 | 2.96 | -0.61 | -0.77 |
| South Sudan | -22.21 | -16.25 | 0 | 2.71 | -1.04 | -1.16 |
| Spain | -23.71 | -9.15 | -1.33 | 2.46 | -1.37 | -0.04 |
| Sri Lanka | -0.95 | -2.92 | 2.08 | 6.18 | 0.26 | 0.68 |
| Sudan | -1.5 | -4.97 | 0.58 | 5.38 | 0.18 | -0.21 |
| Suriname | 4.02 | -3.7 | 1.46 | 4.87 | 0.24 | 0.15 |
| Sweden | 1.71 | -7.4 | -2.3 | 7.37 | 0.87 | -0.9 |
| Switzerland | -1764.12 | -478.99 | -719.09 | -39.82 | -136.04 | -71.69 |
| Syrian Arab Republic | -2.77 | -0.14 | 0.29 | 3.43 | 0.12 | 0.64 |
| Taiwan (Province of China) | -0.97 | -0.44 | 1.75 | 2.65 | 0.12 | -0.52 |
| Tajikistan | -798.31 | -360.5 | -54.82 | 22.27 | -55.92 | -50.7 |
| Thailand | -25.66 | -15.73 | -2.49 | 1.41 | -0.44 | -2.34 |
| Timor-Leste | 2.6 | -0.91 | 1.73 | 2.81 | 0.2 | 0.57 |
| Togo | -1.59 | 0.34 | 0.03 | 1.35 | 0.29 | 0.44 |
| Tokelau | 8.56 | -5.92 | 16.15 | 78.42 | 6.42 | -2.14 |
| Tonga | -3.22 | -16.08 | 2.48 | 9.04 | 0.36 | 0.01 |
| Trinidad and Tobago | -22.01 | -2.05 | -1.8 | 0.38 | -0.52 | -1.37 |
| Tunisia | -5.04 | -1.29 | 0.18 | 3.93 | -0.14 | -0.9 |
| Turkey | -15.85 | -4.13 | -6.03 | 1.77 | -0.49 | -0.75 |
| Turkmenistan | -257.86 | -44.78 | -15.42 | -0.07 | -16.48 | -18.52 |
| Tuvalu | 2.57 | -19.63 | 1.24 | 10.51 | 0.5 | 0.2 |
| Uganda | 1.29 | 2.09 | 2.72 | 2.58 | 0.67 | 0.82 |
| Ukraine | -54.39 | -19.01 | -4.25 | -1.26 | -4.42 | -3.86 |
| United Arab Emirates | -3.56 | -0.91 | -0.34 | 1.43 | -0.12 | 0.61 |
| United Kingdom | -6434.53 | -1790 | -2807.17 | 531.83 | -296.22 | -370.21 |
| United Republic of Tanzania | -0.26 | -0.09 | 1.91 | 2.18 | 0.27 | -0.72 |
| United States of America | -71.89 | -17.53 | -18.49 | 4.34 | -2.92 | -3.75 |
| United States Virgin Islands | 6.23 | 0.08 | 6.47 | 4.95 | 0.45 | -0.3 |
| Uruguay | -376.15 | -76.82 | -38.11 | 61.73 | -12.71 | -29.53 |
| Uzbekistan | -79.44 | -40.52 | -28 | 7.86 | -8.06 | -10.25 |
| Vanuatu | -1.06 | 0.93 | 3.14 | 3.76 | 0.27 | 0.64 |
| Venezuela | -10.79 | 0.65 | 0.31 | 2 | -0.03 | 0.29 |
| Viet Nam | 6.12 | -4.78 | 3.75 | 3.26 | 0.3 | 0.08 |
| Yemen | 0.62 | -4.16 | 0.09 | 2.26 | 0.15 | 0.19 |
| Zambia | -0.11 | -2.16 | 2.98 | 1.52 | 0.18 | 0.29 |
| Zimbabwe | -9.87 | 0.47 | 0.11 | 4.1 | 0.54 | 0.75 |

Table S5 Contribution of Changes in Population Growth, Population Aging, and

Rates of Age-Specific lung cancer mortality to Changes in lung cancer death by gender and region, 1990–2019.

| Location | Gender | Absolute  change | Percent change (%) | Population aging (%) | Population  Growth (%) | Non-GBD risks (%) | all GBD risks (%) | | | | | | |
| --- | --- | --- | --- | --- | --- | --- | --- | --- | --- | --- | --- | --- | --- |
|  |  |  |  |  |  |  | Total | Tobacco | Air  pollution | Occupational  risks | High fasting  plasma glucose | Other environmental risks | Dietary risks |
| Global | Men | 595346 | 75.29 | 75.78 | 65.65 | -1.03 | -40.39 | -24.28 | -6.46 | -7.16 | 1.07 | -1.62 | -1.94 |
|  | Women | 382155 | 139.27 | 42.09 | 42.49 | 10.63 | 4.79 | 0.95 | 0.31 | 0.75 | 2.65 | 0.36 | -0.23 |
| High SDI | Men | 74232 | 26.54 | 200.42 | 97.14 | -3.6 | -193.96 | -118.1 | -21.4 | -41.02 | -1.42 | -6.08 | -5.94 |
|  | Women | 107749 | 93.01 | 49.5 | 29.79 | 15.77 | 4.95 | 0.53 | -2.76 | 1.68 | 4.67 | 0.61 | 0.22 |
| High-middle SDI | Men | 152546 | 53.27 | 116.91 | 52.97 | -5.94 | -63.93 | -40.3 | -12.25 | -5.71 | 0.87 | -3.25 | -3.29 |
|  | Women | 98762 | 129.13 | 49.75 | 25.81 | 7.07 | 17.37 | 11.85 | 1.29 | 1.34 | 2.74 | 0.56 | -0.41 |
| Middle SDI | Men | 281493 | 181.82 | 56.27 | 31.38 | 1.99 | 10.35 | 7.99 | -0.9 | 1.39 | 1.94 | 0.3 | -0.37 |
|  | Women | 130753 | 207.9 | 51.78 | 30.75 | 9.74 | 7.74 | 4.96 | -0.23 | 0.88 | 1.92 | 0.52 | -0.31 |
| Low-middle SDI | Men | 68880 | 130.71 | 44.47 | 51.31 | 3.83 | 0.4 | -1.17 | -3.64 | 3.03 | 2.65 | 0.2 | -0.67 |
|  | Women | 36907 | 236.67 | 36.67 | 37.72 | 13.64 | 11.97 | 3.78 | 2.39 | 1.52 | 2.88 | 0.87 | 0.53 |
| Low SDI | Men | 17926 | 106.74 | 0.13 | 103.92 | 1.93 | -5.98 | -4.44 | -5.14 | 1.52 | 2.23 | -0.08 | -0.07 |
|  | Women | 7803 | 226 | 6.26 | 63.69 | 13.82 | 16.23 | 4.57 | 4.94 | 1.44 | 2.92 | 1.14 | 1.22 |
| Andean Latin America | Men | 1643 | 90.82 | 80.96 | 81.93 | -7.8 | -55.09 | -21.9 | -18.32 | -12.8 | 1.59 | -1.44 | -2.22 |
|  | Women | 1865 | 201.51 | 48.86 | 45.52 | 8.14 | -2.52 | -1.17 | -4.53 | 0.55 | 2.67 | 0.3 | -0.34 |
| Australasia | Men | 1343 | 23.46 | 230.84 | 174.51 | -1.01 | -304.34 | -189.35 | -10.47 | -91.78 | -1.26 | -2.67 | -8.81 |
|  | Women | 2744 | 123.21 | 39.99 | 45.55 | 16.32 | -1.86 | -8.28 | -0.87 | 3.61 | 3.41 | 0.13 | 0.14 |
| Caribbean | Men | 3100 | 73.97 | 70.14 | 52.71 | -1.17 | -21.68 | -15.26 | -5.99 | -0.69 | 2.15 | -0.24 | -1.65 |
|  | Women | 2343 | 146.42 | 48.9 | 32.35 | 7.57 | 11.19 | 6.42 | 0.02 | 1.67 | 3.09 | 0.27 | -0.28 |
| Central Asia | Men | -444 | -3.9 | 644.26 | 824.95 | -167.2 | -1402.02 | -875.13 | -278.27 | -109 | 12.54 | -82.98 | -69.18 |
|  | Women | 302 | 11.06 | 113.36 | 279.83 | -114.92 | -178.27 | -63.15 | -76.17 | -17.4 | 13.63 | -17.07 | -18.11 |
| Central Europe | Men | 9960 | 20.75 | 228.69 | -42.29 | -1.91 | -84.5 | -61.13 | -34.9 | 11.8 | 8.65 | -4.49 | -4.43 |
|  | Women | 13282 | 131.77 | 48.75 | -8.53 | 13.97 | 45.81 | 30.7 | 2.69 | 2.66 | 5.73 | 2.4 | 1.63 |
| Central Latin America | Men | 8421 | 105.71 | 87.55 | 58.5 | 4.53 | -50.58 | -34.22 | -10.91 | -1.5 | -1.23 | -1.57 | -1.15 |
|  | Women | 6789 | 166.82 | 69.24 | 44.75 | 6.08 | -20.08 | -13.58 | -5.76 | 0.25 | -0.13 | -0.43 | -0.43 |
| Central Sub-Saharan Africa | Men | 2400 | 84.24 | -9.67 | 144.71 | -3.5 | -31.55 | -14.67 | -13.86 | -3.22 | 1.35 | -0.76 | -0.39 |
|  | Women | 1070 | 187.2 | 2.8 | 80.46 | 11.27 | 5.47 | 0.71 | -0.15 | 1.17 | 2.16 | 0.56 | 1.02 |
| East Asia | Men | 353039 | 192.13 | 68.88 | 16.55 | 1.19 | 13.38 | 10.51 | -0.02 | 1.4 | 1.6 | 0.4 | -0.51 |
|  | Women | 160648 | 198.45 | 65.1 | 18.29 | 6.94 | 9.65 | 7.58 | 0.01 | 0.9 | 1.3 | 0.53 | -0.67 |
| Eastern Europe | Men | -18063 | -22.26 | 144.97 | -33.17 | -23.9 | -187.91 | -121.98 | -35.38 | -10.25 | -1.78 | -9.4 | -9.12 |
|  | Women | -1345 | -7.93 | 289.58 | -86.67 | -156.92 | -145.98 | -9.14 | -88.51 | -20.98 | 1.81 | -12.74 | -16.42 |
| Eastern Sub-Saharan Africa | Men | 4071 | 96.52 | -7.81 | 115.27 | 1.59 | -9.06 | -5.21 | -6.61 | 1.87 | 1.61 | -0.21 | -0.51 |
|  | Women | 2164 | 218.27 | 4.74 | 65.64 | 15.6 | 14.03 | 2.89 | 5.27 | 2.28 | 1.75 | 0.93 | 0.91 |
| High-income Asia Pacific | Men | 40364 | 110.75 | 119.87 | 10.4 | 2.16 | -32.43 | -28.42 | -1.24 | -0.2 | -0.79 | -0.16 | -1.62 |
|  | Women | 20235 | 145.29 | 92.53 | 9.12 | 9.34 | -11.01 | -12.03 | 1.05 | -0.68 | 0.89 | 0.3 | -0.54 |
| High-income North America | Men | 16576 | 15.06 | 290.55 | 197.13 | -7.83 | -379.85 | -236.02 | -40.51 | -78.09 | -2.92 | -10.61 | -11.7 |
|  | Women | 42413 | 69.22 | 49.19 | 48.52 | 13.05 | -10.76 | -9.83 | -7.07 | 1.01 | 5.57 | 0.07 | -0.51 |
| North Africa and Middle East | Men | 31742 | 125.11 | 45.19 | 71.8 | -0.79 | -16.2 | -10.87 | -3.67 | -3.06 | 2.67 | -0.44 | -0.83 |
|  | Women | 11222 | 268.89 | 29.32 | 41.89 | 12.97 | 15.81 | 5.77 | 4.2 | 0.66 | 4.24 | 0.84 | 0.1 |
| Oceania | Men | 709 | 152.65 | 16.77 | 76.36 | 4.46 | 2.41 | -2.39 | -0.72 | 1.82 | 3.51 | 0.14 | 0.05 |
|  | Women | 208 | 192.23 | 13.02 | 66.38 | 9.21 | 11.4 | 2.89 | 1.73 | 1.21 | 4.5 | 0.48 | 0.59 |
| South Asia | Men | 51559 | 144.82 | 45.71 | 53.42 | 4.4 | -3.54 | -5.86 | -2.73 | 3.05 | 2.54 | -0.06 | -0.48 |
|  | Women | 25227 | 347.57 | 32.85 | 34.41 | 15.54 | 17.21 | 3.89 | 6.32 | 1.3 | 3.32 | 1.07 | 1.31 |
| Southeast Asia | Men | 52911 | 133.94 | 59.31 | 44.15 | 2.66 | -6.1 | -1.58 | -8.54 | 2.28 | 2.55 | 0 | -0.81 |
|  | Women | 27799 | 193.7 | 49.55 | 33.64 | 14.22 | 2.58 | 1.03 | -3.12 | 1.27 | 3.05 | 0.31 | 0.04 |
| Southern Latin America | Men | 1847 | 16.45 | 197.36 | 197.84 | -29.06 | -266.14 | -199.75 | -38.17 | -13.4 | 5.67 | -6.36 | -14.13 |
|  | Women | 4040 | 160.3 | 31.94 | 31.56 | 12.49 | 24.01 | 13.9 | 1.31 | 3.41 | 4.59 | 0.82 | -0.02 |
| Southern Sub-Saharan Africa | Men | 2860 | 69.39 | 53.83 | 78.38 | 6.99 | -39.2 | -25.52 | -9.74 | -5.14 | 2.97 | -0.76 | -1.01 |
|  | Women | 2017 | 136.9 | 40.78 | 45.75 | 15.02 | -1.54 | -5.56 | -0.79 | -0.65 | 4.11 | 0.59 | 0.76 |
| Tropical Latin America | Men | 10775 | 93.63 | 93.21 | 57.29 | 7.08 | -57.59 | -38.11 | -13.27 | -1.9 | -0.79 | -1.48 | -2.04 |
|  | Women | 11191 | 254.76 | 50.45 | 31.17 | 14.54 | 3.84 | 2.2 | -2.63 | 1.72 | 1.95 | 0.69 | -0.09 |
| Western Europe | Men | 13783 | 8.62 | 475.55 | 168.48 | -14.45 | -529.58 | -308.26 | -77.82 | -112.65 | -0.79 | -18.31 | -11.75 |
|  | Women | 44746 | 105.84 | 33.79 | 16.29 | 16.58 | 33.35 | 21.32 | -1.52 | 4.1 | 6.14 | 2.13 | 1.18 |
| Western Sub-Saharan Africa | Men | 6749 | 124.82 | -19.64 | 104.99 | 8.37 | 6.26 | 2.45 | 0.17 | 0.07 | 2.7 | 0.53 | 0.34 |
|  | Women | 3197 | 210.4 | -7.34 | 77.07 | 18.3 | 11.97 | 1.37 | 5.35 | 0.22 | 2.87 | 1.15 | 1.01 |
