# Supplementary figures and images for: Epidemiological and demographic drivers of lung cancer mortality from 1990 to 2019: results from the global burden of disease study 2019
Source: Front Public Health. 2023 May 5;11:1054200. doi: 10.3389/fpubh.2023.1054200 (PMC10196253; doi:10.3389/fpubh.2023.1054200)

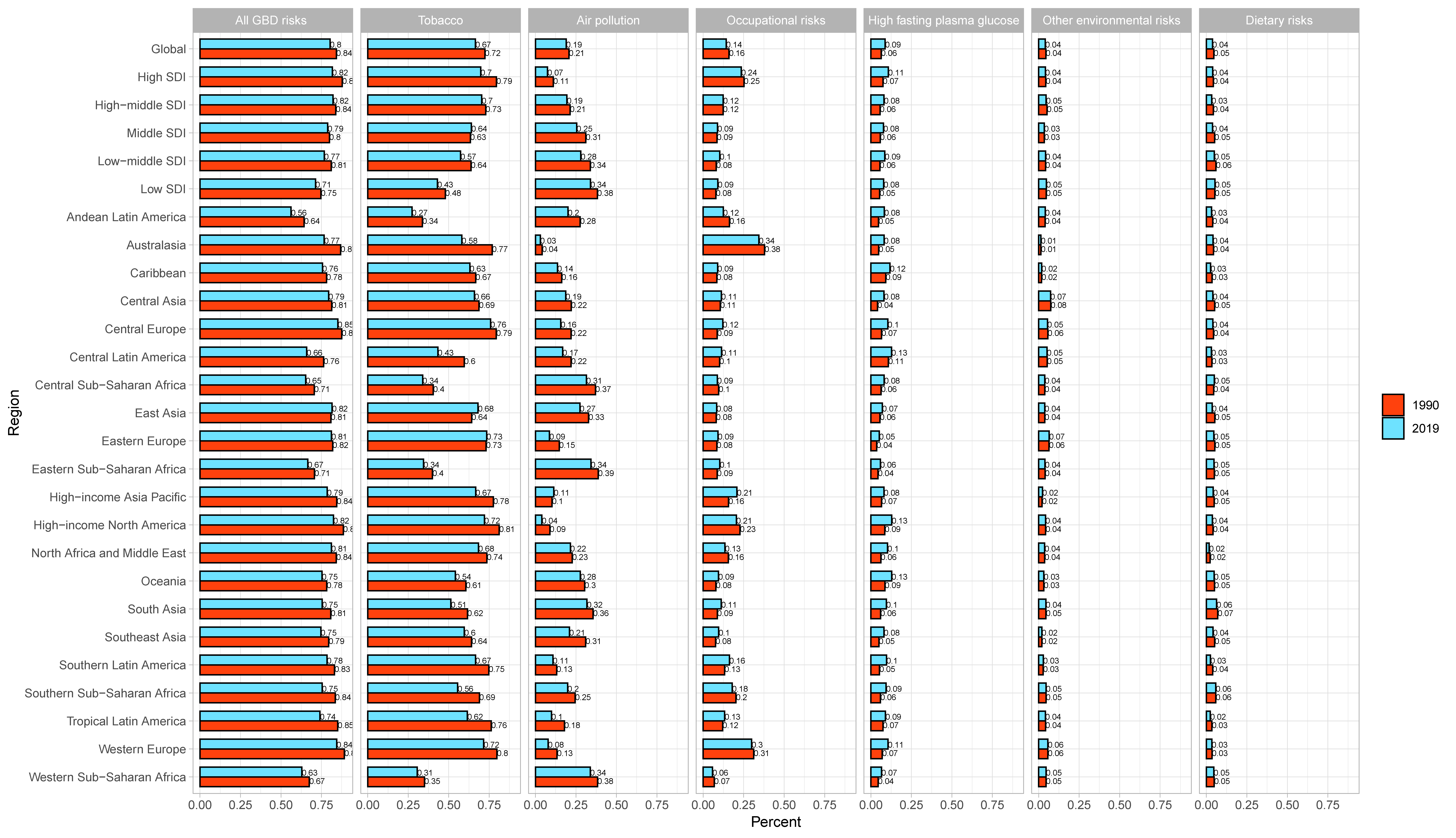

Supplement: Supplementary file 2 [file Image_1.tif]

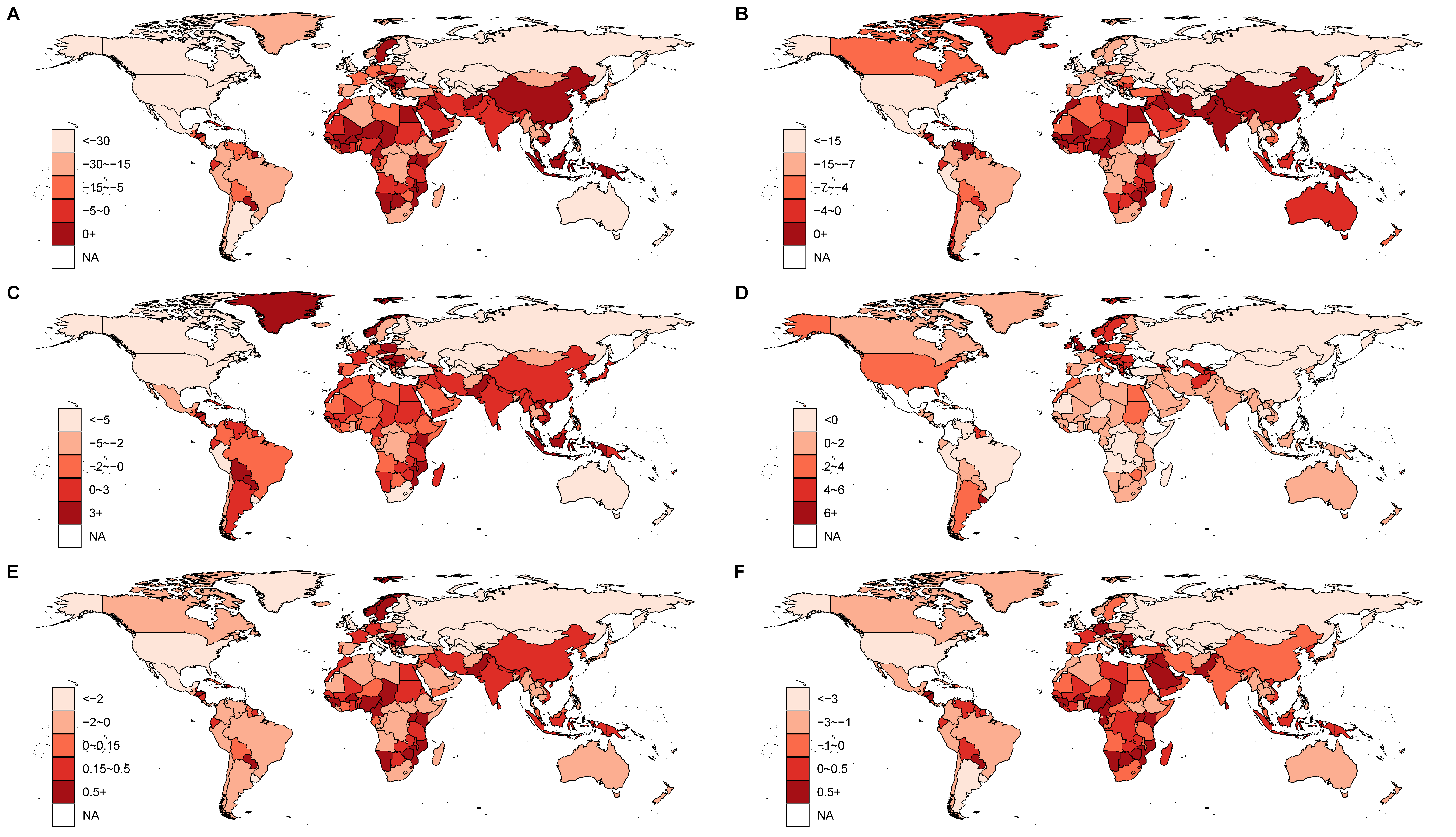

Supplement: Supplementary file 3 [file Image_2.tif]

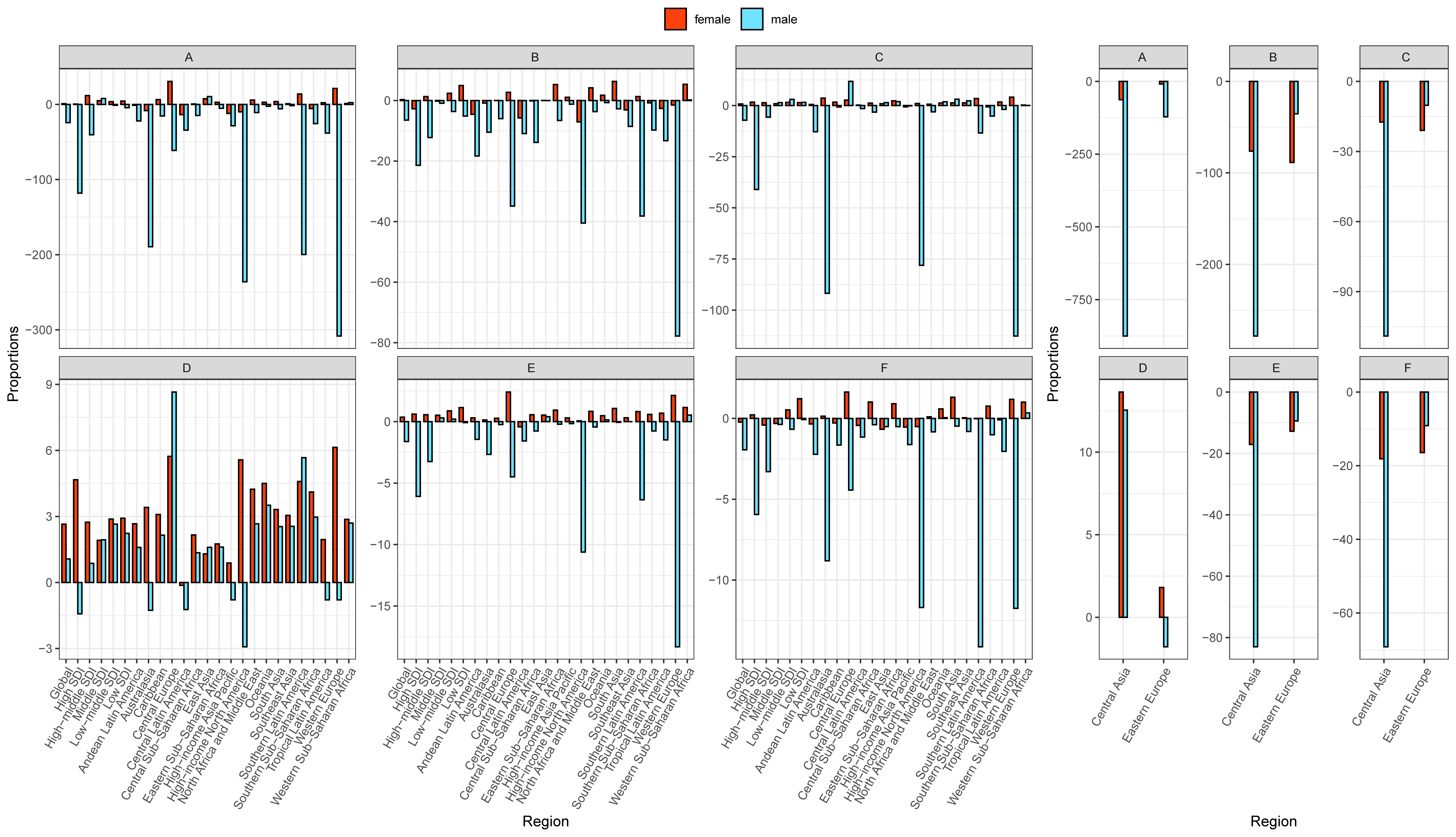

Supplement: Supplementary file 4 [file Image_3.tif]

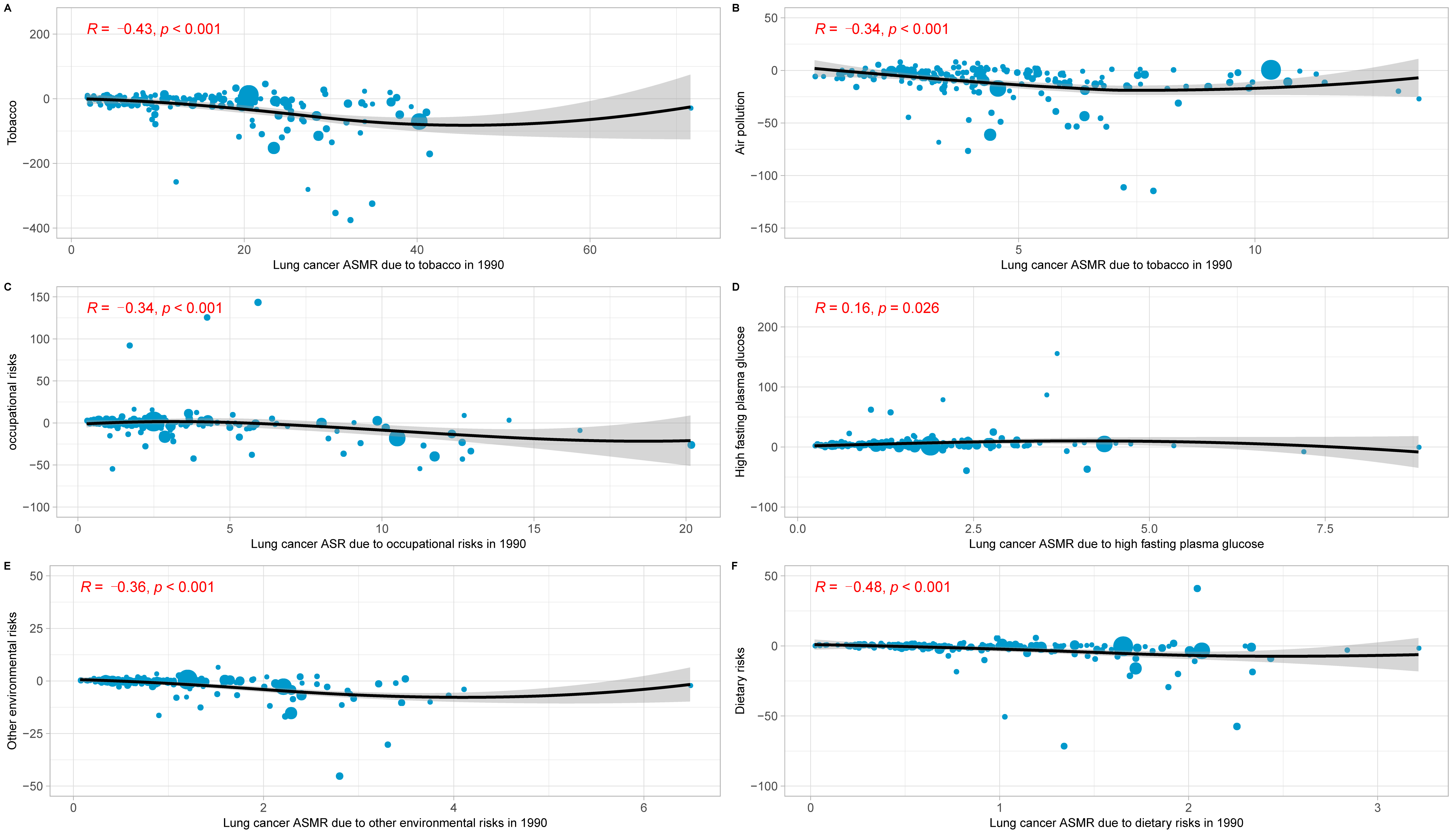

Supplement: Supplementary file 5 [file Image_4.tif]

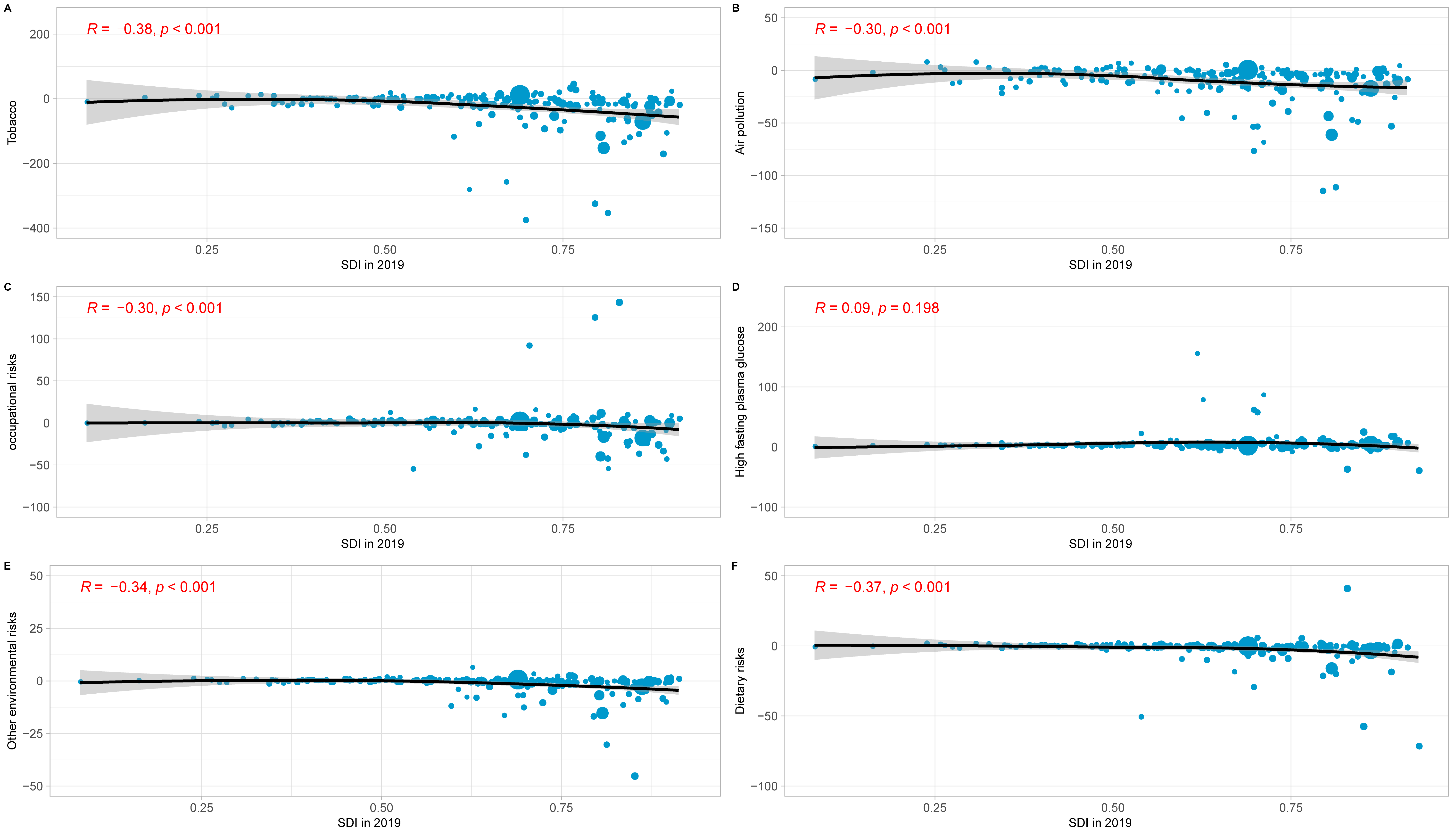

Supplement: Supplementary file 6 [file Image_5.tif]

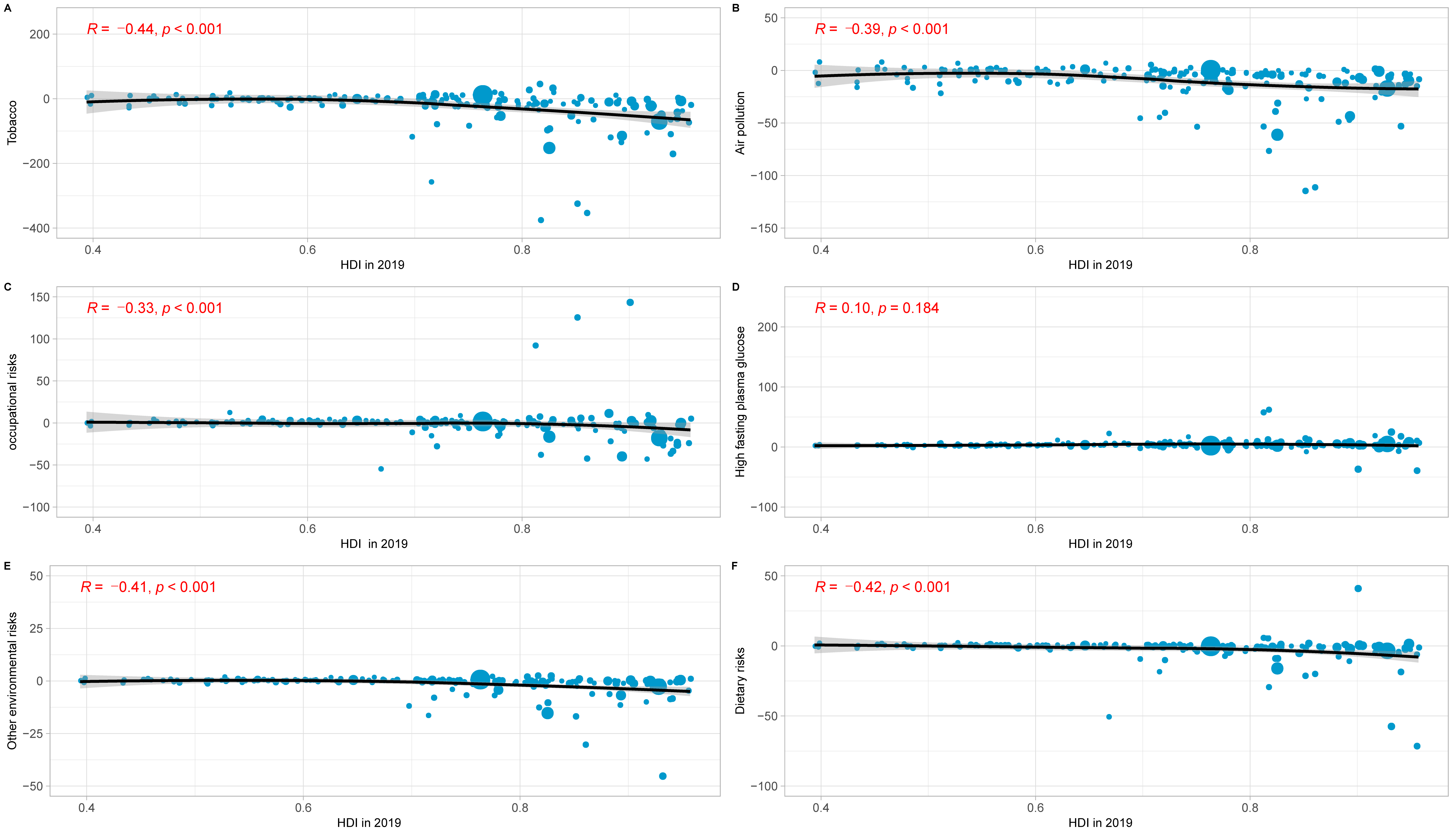

Supplement: Supplementary file 7 [file Image_6.tif]
