# Supplementary material for: Epidemiological and demographic drivers of lung cancer mortality from 1990 to 2019: results from the global burden of disease study 2019
Source: Front Public Health. 2023 May 5;11:1054200. doi: 10.3389/fpubh.2023.1054200 (PMC10196253; doi:10.3389/fpubh.2023.1054200)
Supplement: Supplementary file 8 [file Data_Sheet_1.docx]

**Supplementary Figures and Tables**

**SUPPLEMENTARY FIGURE S1**

Percentage of lung cancer mortality attributable to GBD risk factors in 2019 at global and regional level (generated from data available from http://ghdx.healthdata.org/gbd-results-tool).

**SUPPLEMENTARY FIGURE S**2

Contribution of changes in age-specific lung cancer mortality from specific level 2 risks factors to changes in lung mortality, 1990–2019 at national level, using 1990 as the reference year. (A) Tobacco, (B) Air pollution, (C) Occupational risks, (D) High fasting plasma glucose, (E)other environmental factors, and (F) dietary risks.

**SUPPLEMENTARY FIGURE S3**

Contribution of changes in age-specific lung cancer mortality from specific level 2 risks factors to changes in lung mortality, 1990–2019 at regional level by gender, using 1990 as the reference year, (A) Tobacco, (B) Air pollution, (C) Occupational risks, (D) High fasting plasma glucose, (E)other environmental factors, and (F) dietary risks.

**SUPPLEMENTARY FIGURE S**4

Percentage Change in lung cancer mortality owing to changes in age-specific lung cancer mortality due to specific level 2 risk factors, according to lung cancer ASMR in 1990.

**SUPPLEMENTARY FIGURE S5**

Percentage Change in lung cancer mortality owing to changes in age-specific lung cancer mortality due to specific level 2 risk factors, according to SDI in 2019.

**SUPPLEMENTARY FIGURE S6**

Percentage Change in lung cancer mortality owing to changes in age-specific lung cancer mortality due to specific level 2 risk factors, according to HDI in 2019.

**Supplementary Tables**

**Supplementary Table 1**

Regional EAPC of all-age and age-standardized lung cancer mortality from 1990 to 2019

**Supplementary Table 2**

Contribution of changes in population growth, population aging, and rates of age-specific lung cancer mortality to changes in lung cancer deaths by region, 1990–2019

**Supplementary Table 3**

Contribution of Changes in population growth, population aging, and rates of age-specific lung cancer mortality to changes in lung cancer death by country/territory, 1990–2019

**Supplementary Table 4**

Contribution of specific GBD risks to changes in lung cancer deaths by country/territory, 1990–2019

**Supplementary Table 5**

Contribution of Changes in Population Growth, Population Aging, and Rates of Age-Specific lung cancer mortality to Changes in lung cancer death by gender and region, 1990–2019
